# Trehalosemodulates OVRAS to improve oxidative stress and apoptosis in KGN cells and ovaries of PCOS mice

**DOI:** 10.1186/s13048-023-01337-5

**Published:** 2024-01-09

**Authors:** Shasha Gao, Juan Wang, Lun Wei, Chao Luo, Fei Qian, Le Bo, Caiping Mao

**Affiliations:** Reproductive Medicine Center, The First Affiliated Hospital of Soochow University, 899 Pinghai Rd, Suzhou, Jiangsu 215000 China

**Keywords:** Polycystic ovary syndrome, Trehalose, Renin-angiotensin system, KGN

## Abstract

The etiology of polycystic ovary syndrome (PCOS) is complex and variable, and there is no exact cause or good treatment method. Most of the methods of hormones are used to temporarily meet the needs of patients. Experimental evidence has shown that trehalose has, anti-apoptotic, anti-oxidative, glucose-lowering, and insulin resistance effects. However, whether trehalose has a therapeutic effect on PCOS is unknown. It has been reported that the ovarian renin-angiotensin system (OVRAS) is involved in the development of PCOS, but it has not been fully elucidated. This study aims to explore the effect of trehalose on PCOS and elucidate the related OVRAS mechanism. We first observed that body weight, estrous cycle, ovarian follicles at all levels, glucose tolerance, serum hormones, and insulin resistance were improved by trehalose treatment in the PCOS mouse model. Moreover, trehalose treatment also ameliorated ovarian oxidative stress and apoptosis in PCOS mice, as determined by TUNNEL apoptosis staining, total SOD in ovarian homogenate, and WB assay. OVRAS mainly involves two classic pathways, namely the ACE/AngII/AT1R/AT2R, and ACE2 / Ang1-7/ MASR, Which play different functions. In PCOS mouse ovaries, we found that ACE/AngII/AT1R was up-regulated and ACE2/Ang1-7/MASR and AT2R were down-regulated by PCR and WB experiments, However, trehalose treatment changed its direction. In addition, we also found that trehalose ameliorated DHEA-induced oxidative stress and apoptosis in KGN by PCR and WB experiments, mainly by down-regulating ACE/AngII/AT1R. Our study shows that trehalose improves symptoms of PCOS mainly by down-regulating ACE/AngII/AT1R, revealing a potential therapeutic target for PCOS.

**Supplementary Information:**

The online version contains supplementary material available at 10.1186/s13048-023-01337-5.

## Introduction

Polycystic ovary syndrome (PCOS) is one of the most common endocrine and metabolic disorders in women of reproductive age. According to statistics, it affects up to 4%-20% of women of reproductive age worldwide [1]. The revised 2018 ESHRE/ASRM (Rotterdam) diagnostic criteria for adult women: Any individual presenting with two of three features, (1) oligoovulation/anovulation (OA), (2) hyperandrogenism (HA), and (3) polycystic ovary (PCOM), but first excluding other potentially similar conditions, can be diagnosed with polycystic ovary syndrome. In adolescents, only oligoanovulation and hyperandrogenism are diagnosed. Ultrasound is not recommended for diagnosis [2]. This syndrome can lead to insulin resistance, obesity, infertility, miscarriage [3, 4], pre-eclampsia, neonatal complications [5], and other long-term complications such as endometrial cancer, type II diabetes [6], depression [7], and cardiovascular problems [8, 9]. A series of symptoms are mainly caused by hyperandrogenism and insulin resistance. Hyperandrogenism and insulin resistance promote each other, forming a vicious circle and promoting the further development of the disease [10]. The pathogenesis of this disease is still unclear, and the pathophysiological mechanisms are complex and diverse, including oxidative stress [11, 12], apoptosis [13], chronic inflammation [14], HPO axis abnormality immune dysregulation [15], etc. At present, there is no single effective treatment for this disease. At present, it mainly relies on the symptoms and needs of patients for temporary empirical medication. There are also many difficulties in medication process, such as adverse reactions, low compliance of patients with long-term drug therapy, low efficacy, and contraindications in some cases. Therefore, it is urgent to find new drugs or molecular targets for treatment [16]. Trehalose is a disaccharide of glucose, a naturally stable non-toxic, and non-reducing bioactive sugar. It was initially considered an energy storage but later found to have special cytoprotective effects. Trehalose is widely present in various natural foods and is also used as a food additive, which is approved for human use according to FDA requirements [17, 18]. In the past decade, trehalose has attracted special attention. It has been found to have various pharmacological effects, including anti-inflammatory, anti-oxidative [19], antiviral [20], and anti-tumor [21] effects. It has therapeutic effects in experimental models of various diseases [22, 23]. The non-toxicity of trehalose allows it to be administered in the human body for a long time. Although it is broken down into two glucose molecules in the small intestine, oral trehalose molecules have been shown to treat various diseases. The mechanism is elusive and may be related to the potential changes in the tissue structure and secretion of metabolites by trehalose [24, 25]. Trehalose has received much attention for its ability to regulate glucose homeostasis, increase insulin sensitivity, and reduce insulin resistance [26], and may serve as a therapeutic agent for diabetes [27]. Second, trehalose also reduced atherosclerosis, dyslipidemia, hepatic steatosis, and cardiometabolic disease burden in diet-induced and genetic insulin and glucose tolerance models [28, 29]. However, whether trehalose exerts anti-apoptosis, anti-oxidation, insulin resistance reduction and other effects in PCOS and the underlying molecular mechanisms are still unknown.

Classical RAS can be divided into two major pathways: ACE/AngII/AT1R/AT2R and ACE2/Ang1-7/MasR. It has been believed that RAS is closely related to the occurrence and development of cardiovascular, renal, and endocrine diseases. In recent years, it has been found that RAS plays a role in regulating cell growth, apoptosis and autophagy, oxidative stress, inflammatory response, and neovascularization, and local RAS has been found in the ovary [30, 31], uterus, testis [32, 33], placenta and other tissues. ACE/AngII/AT1R mainly promotes vasoconstriction, cell growth, proliferation and differentiation, and inflammatory response. ACE2/Ang1-7/MasR and AT2R play a role in vasodilation, glucose and lipid metabolism regulation, anti-proliferation, and anti-oxidative stress [34, 35]. The ovarian renin-angiotensin system (OVRAS) is known to be involved in ovarian steroidogenesis, follicular development (including oocyte maturation), ovulation, and follicular atresia [36, 37], and the same molecule may play different roles in different species [38]. OVRAS has been linked to several reproductive disorders. Such as PCOS, OHSS, ovarian cancer, and infertility [39]. In PCOS, components in OVRAS are also associated with the number of mature follicles, insulin resistance, and androgen levels [31]. Existing studies have shown that the ovarian renin-angiotensin system (OVRAS) may be involved in the occurrence and development of PCOS [40], but the specific mechanism has not been fully elucidated. The present study investigated the effects of trehalose on the ovarian function of PCOS mice and KGN cells and the mechanism of local RAS, which may provide potential strategies for treating of PCOS.

## Materials and methods

### Animals and feeding

Forty-five female C57BL/6 mice of SPF grade, aged 3 weeks (9-11 g), license number: SCXK (Zhejiang) 2019-0004, qualification certificate number: 20210802Abzz0105000375, were purchased from Hangzhou Ziyuan Laboratory Animal Technology Co., LTD. Animals were housed in the SPF-grade experimental animal room of Soochow University, Jiangsu Province, and mice were housed in a temperature-controlled room (20–26 °C) with normal dark light (12 h: 12 h) cycles with access to food and water at any time. After 7 days of adaptation, mice were randomly divided into control group(N) (*n* = 15), PCOS group(P) (*n* = 15), and trehalose group(S) (*n* = 15).

### Modeling dose information

For animal modeling experiments, mice were randomly divided into three groups. Control group: Mice were fed a normal diet for 24 days, plus injection of DHEA solvent such as soybean oil into the back of the neck for the first 21 days. PCOS group: Mice were fed an HFD (High-fat diet) (consisting of 61.5% regular diet, 12% lard, 5% sucrose, 5% milk powder, 5% peanut, 10% egg, 1% sesame oil and 0.5% salt) and injected with DHEA (6 mg/kg/d, dissolved in DMSO and fused with soybean oil) on the back of neck for 21 days, followed by HFD for the remaining 3 days. Trehalose group: In addition to the intervention in the PCOS group, 2% trehalose (R8530; Yuanye, China) were fed with water for 24 days (trehalose dissolved in the drinking water of mice).

### Weight changes and vaginal smears

From the beginning to the end of modeling, the weight change of mice in each group was recorded every week. The vaginal smear was performed simultaneously during the last 10 days of modeling: Vaginal secretions of mice were taken out by dipping a cotton swab with normal saline and evenly spread on the glass slide. After natural air drying, the mice were fixed with absolute methanol for 15 min, stained with hematoxylin for 3 min, and slowly rinsed under running water for 5 min. After natural air drying, the mice were stained with eosin for 5 min and slowly rinsed under water for 3 min. Moreover, determine the period of estrus.

### Glucose tolerance test

After completing 24 modeling days, mice in each group were fasted for 14 to 16 h, and initial blood glucose was measured. Glucose (3 g/kg) was administered by intraperitoneal injection, and blood glucose concentrations were measured from the tail vein of mice 15,30,60,90, and 120 min after glucose administration using a glucometer.

### Detection by ELISA

Blood was collected from the abdominal aorta of mice and stored at room temperature (25 ± 2 °C) for 2–3 h. Serum was obtained by centrifugation at 4000 rpm for 10 min at room temperature. Serum insulin, estrogen, androgen levels were measured byEnzyme-linked immunoassay. Then ovarian tissue was obtained for the preparation of ovarian tissue homogenate, and the levels of AngII and Ang1-7 were detected by Enzyme-linked immunoassay.

### HE staining

Ovaries were fixed in 4% paraformaldehyde for 48 h, followed by stepwise dehydration and subsequent embedding in paraffin. Paraffin-embedded tissue sections (5 μm) were retained after five serial sections were discarded, deparaffinized, hydrated, stained with hematoxylin and eosin (H&E), sealed and stored in neutral resin, and photographed with an inverted microscope.

### Ovarian Tunnel apoptotic staining and total SOD assay

Mouse ovaries and ovarian tissue homogenates were obtained according to the Tunnel kit and total SOD activity detection kit (S0101S, Beyotime, China), respectively.

### Cell culture and processing

The human ovarian granulosa cell line was received from Professor Chen Li, Reproductive Medicine Center, General Hospital of Eastern Theater Command of PLA (Jinling Hospital Affiliated with Medical School of Nanjing University). KGN cells were cocultured with DMEM/F12 (Hyclone, USA), 10% fetal bovine serum (FBS, Invitrogen Gibco, USA), and 1% dual antibody and maintained in a 5%CO2, 37 °C environment.

KGN cells were seeded in 6-well plates with approximately 10^5^ cells per well.Control group: One thought of DMSO solvent was added In cell culture medium. PCOS group: According to the relevant literature, the cells were treated with dehydroepiandrosterone (DHEA dissolved in DMSO) at a concentration of 10^−5^ mol/l for 48 h. Trehalose group: In addition to the treatment of the PCOS group, KGN cells were treated with trehalose at a concentration of 10^–5^ for 24 h.

### EDU, ROS, and JC-1 assays

After incubation with the EDU (Beyotime, China), ROS (Beyotime, China), and JC-1 (Beyotime, China) kits for the corresponding time, the cells were washed twice, and the results were observed under a fluorescence microscope and recorded.

### Real-time quantitative chain reaction (qPCR)

KGN cells and mouse tissues were prepared for lysis (lysate added in the Kit), RNA was extracted with RNA-Quick Purification Kit ES (Yixian Bio, RN001), and the corresponding concentrations were measured with Qubit®RNA HS Assay Kits (Thermo, USA). According to the manufacturer’s instructions, cDNA was synthesized using a reverse transcription kit (RR047A, TakaRa). RT-qPCR was performed using a fluorescent quantitative PCR instrument (Step One Plus), Power Up SYBR Green Master Mix (Applied Biosystems, USA), Free-RNAase H2O, forward and reverse primers, and PCR conditions were as follows: 40 cycles were performed at 50 °C for 2 min and 95 °C for 2 min: 3 s at 95 °C and 30 s at 60 °C. The sequences of all primers are shown in Table 1, and the sequences are arranged according to 5’➔3’. GAPDH was used as an internal control. All experiments were repeated at least three times to ensure the reliability of the experimental data. Relative gene expression (RGE) was calculated as RGE = 2 ^− ΔΔCt^.

**Table 1:**
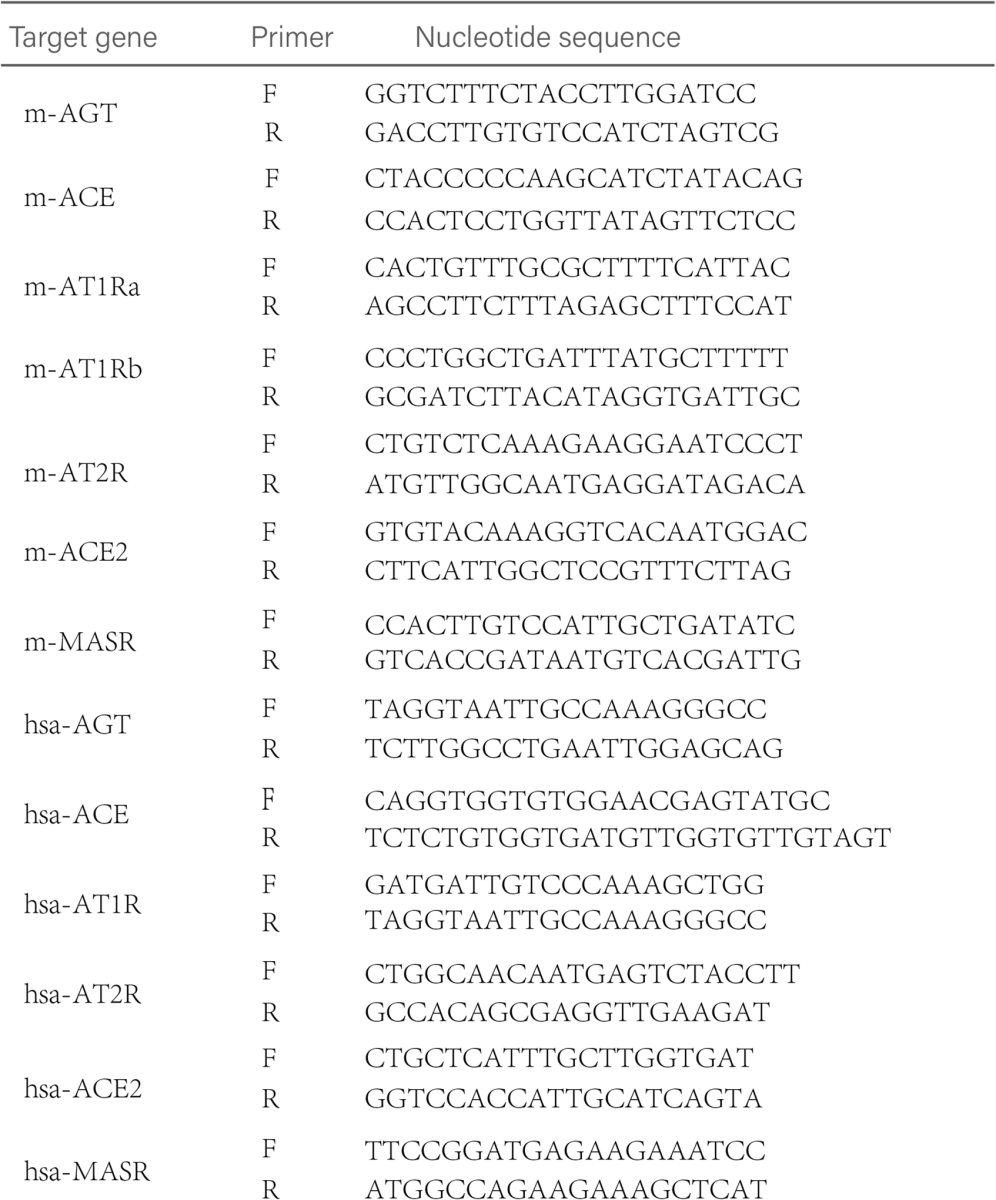
Primers used in this experiment

### Western blot

Protein imprinting method to detect the AGT, ACE/AT1R/AT2R, ACE2 / MASR, BAX, BCL 2, Cleaved caspase - 9, SOD2 expression. The prepared lysate: PIPA: PMSF (100 mM):A: B = 100:1 was mixed and added to mouse ovary and KGN cells for full lysis. The total protein was collected and the protein concentration was determined using the BCA Protein Quantification Kit Enhanced (Biyantian, P0010S). The same amount (10 μg) was separated by electrophoresis on a 10%SDS-PAGE gel and transferred to a PVDF transfer membrane. The membranes were blocked in 5% skim milk for 2 h at room temperature, the three sides of the membranes were washed, and the primary antibody (AGT, 1:1000; ACE1, 1: 1000; AT1R, 1: 1000; AT2R, 1: 1000; ACE2, 1: 1000; MASR, 1: 1000; SOD2, 1: 1000; BAX, 1: 1000; BCL-2, 1: 1000; Cleaves caspase-9, 1: 1000; GAPDH, 1: 10000) After overnight incubation with primary antibody at 4°, membranes were washed three times, and the corresponding rabbit secondary antibody (1:1000) or mouse secondary antibody (1:1000) was diluted with TBST and incubated with the secondary antibody for 1 h at room temperature on a shaker for autoradiography. The results were analyzed by Image J software.

### Statistical analysis

Data were analyzed using GraphPad Prism 8.0 (GraphPad Software, USA) and presented as mean ± standard deviation. Fluorescence intensity and Western blot results were analyzed using Image J software for gray values. The data were normally distributed and described by mean plus or minus standard deviation (x + s). One-way analysis of variance was used to compare the data between groups after the normal distribution and homogeneity of variance test, and the LSD method was used for multiple comparisons. If the data variance was not uniform, Tamhane’s T2 method in the non-parametric test was selected. *P* < 0.05 was statistically significant, and *P* < 0.01 was statistically significant.

## Results

### Body weight and estrous cycle changes

**Fig. 1:**
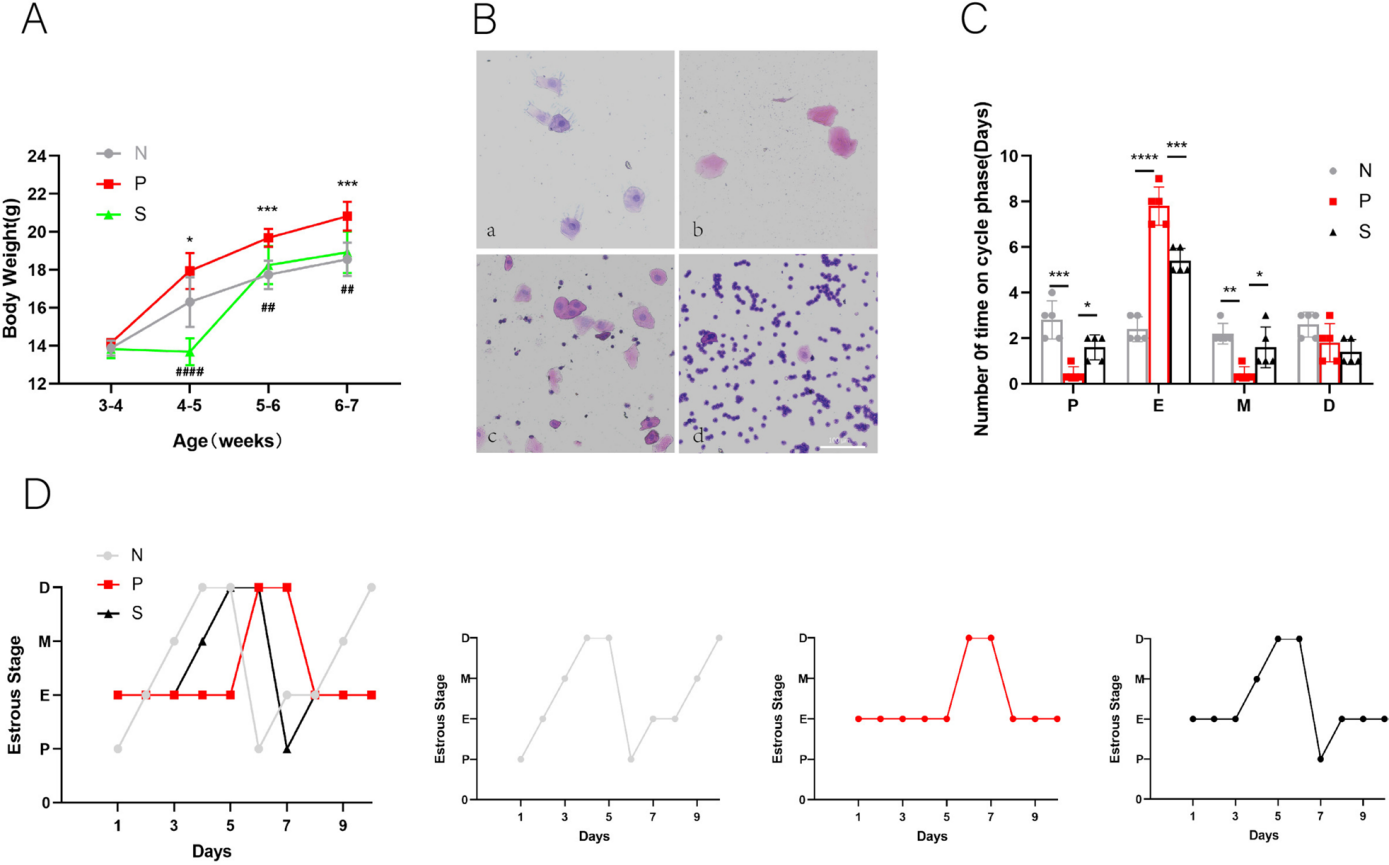
Effect of trehalose on body weight and estrous cycle in PCOS mice. **A** Line chart of weight change of mice in each group during modeling, where * is the mark of comparison between N group and P group, # is the mark of comparison between P group and S group; **B** pictures of four phases of the mouse estrous cycle; **C** Broken line statistics of estrous cycle changes of mice in each group; **D** Histogram of the number of days in proestrus (P), estrus (E), metestrus (M), and diestrus (D) in each group of mice. the number of days with 0 was calculated as 0.3. N stands for control, P for PCOS, and S for trehalose. Data are presented as M + SD, N group compared with P group, P group compared with S group, *P < 0.05, **P < 0.01, ***P < 0.001, ****P < 0.0001

The body weight of mice increases and the estrus cycle disorder is a key indicator to reflect the success of the PCOS model. Our modeling started at 3–4 weeks of age, and the body weight of the mice was measured once a week. We found that the PCOS group had a rapid increase in body weight compared with the Control group (*P* < 0.05), while the body weight decreased after trehalose treatment (Fig. 1A) (*P* < 0.05). The normal estrous cycle of mice is 4–6 days and is divided into proestrus (P) (Fig. 1Ba), estrus (E) (Fig. 1Bb), metestrus (M) (Fig. 1Bc), and diestrus (D) (Fig. 1Bd). Proestrus: Abundant nucleated epithelial cells, few keratinized cells. Estrus: numerous keratinocytes, few nucleated epithelial cells.Metestrus: nucleated cells, keratinocytes, and leukocytes coexist. Diestrus: Full field of white blood cells. Vaginal smears were taken 10 days before the end of modeling to observe the changes in the estrus cycle in mice. We found that PCOS mice had a disordered, prolonged, or even completely disordered estrus cycle (> 6 days), mainly manifested as a longer time in estrus. In contrast, the estrus cycle was shorter after trehalose treatment than that of the PCOS group. Proestrus and anestrus begin to appear (Fig. 1C-D).

### Relevant hormone and biochemical tests were performed

**Fig. 2:**
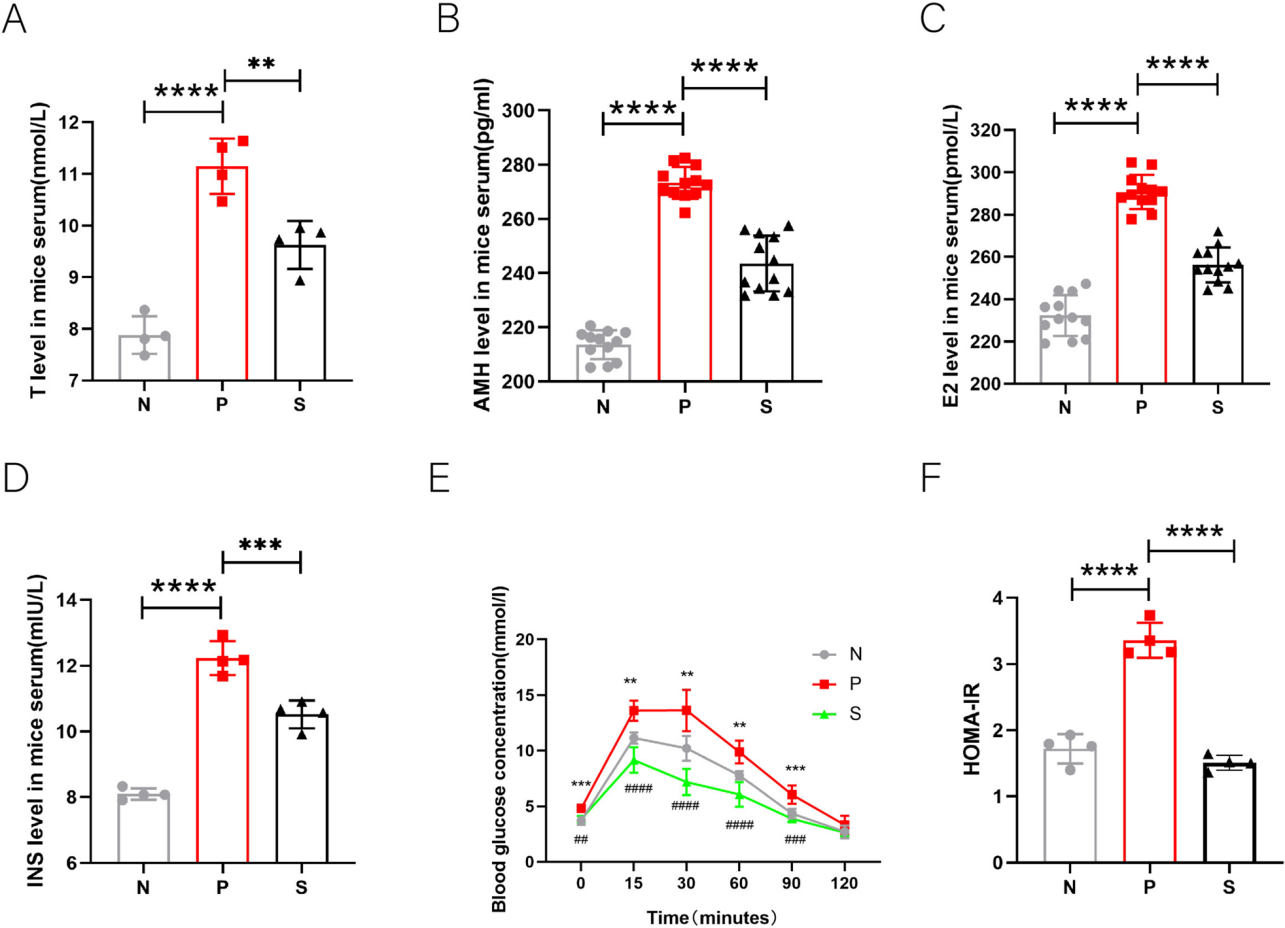
Effects of trehalose on hormones and blood glucose in PCOS mice. **A** Changes of serum T in each group of mice. **B** The changes of serum AMH in each group; **C** changes of serum E2 in each group; **D** changes of serum INS in each group; **E** Glucose tolerance test (OGTT) of mice in each group, where * is the mark of comparison between N group and P group, # is the mark of comparison between P group and S group; **F** Statistical plot of insulin resistance of mice in each group, HOMA-IR = FBG × FINS/22.5. N stands for control,P for PCOS, and S for trehalose. Data are presented as M + SD, N group compared with P group, P group compared with S group, *P < 0.05, **P < 0.01, ***P < 0.001, ****P < 0.0001

Many studies have shown that hormonal changes, such as elevated T, INS.E2, AMH, etc., accompany PCOS. Androgen combined with insulin resistance is the leading cause of many disease complications. In addition, most PCOS patients have impaired glucose tolerance (OGTT) due to insulin resistance and elevated insulin, so we further performed an OGTT test. It can be seen from the figure that the PCOS model was successful in terms of hormones, including the increase of T and insulin resistance (Fig. 2A&F), accompanied by the increase of AMH and E2 (Fig. 2B&C) (*P* < 0.001). The OGTT test also proved that the glucose tolerance of the PCOS group (Fig. 2E) was decreased (*P* < 0.05). However, after trehalose treatment, the T, INS, E2, AMH, and HOMA-IR levels decreased, and the impaired glucose tolerance was also repaired (*P* < 0.01).

### Size of ovaries and number of follicles (HE staining)

**Fig. 3:**
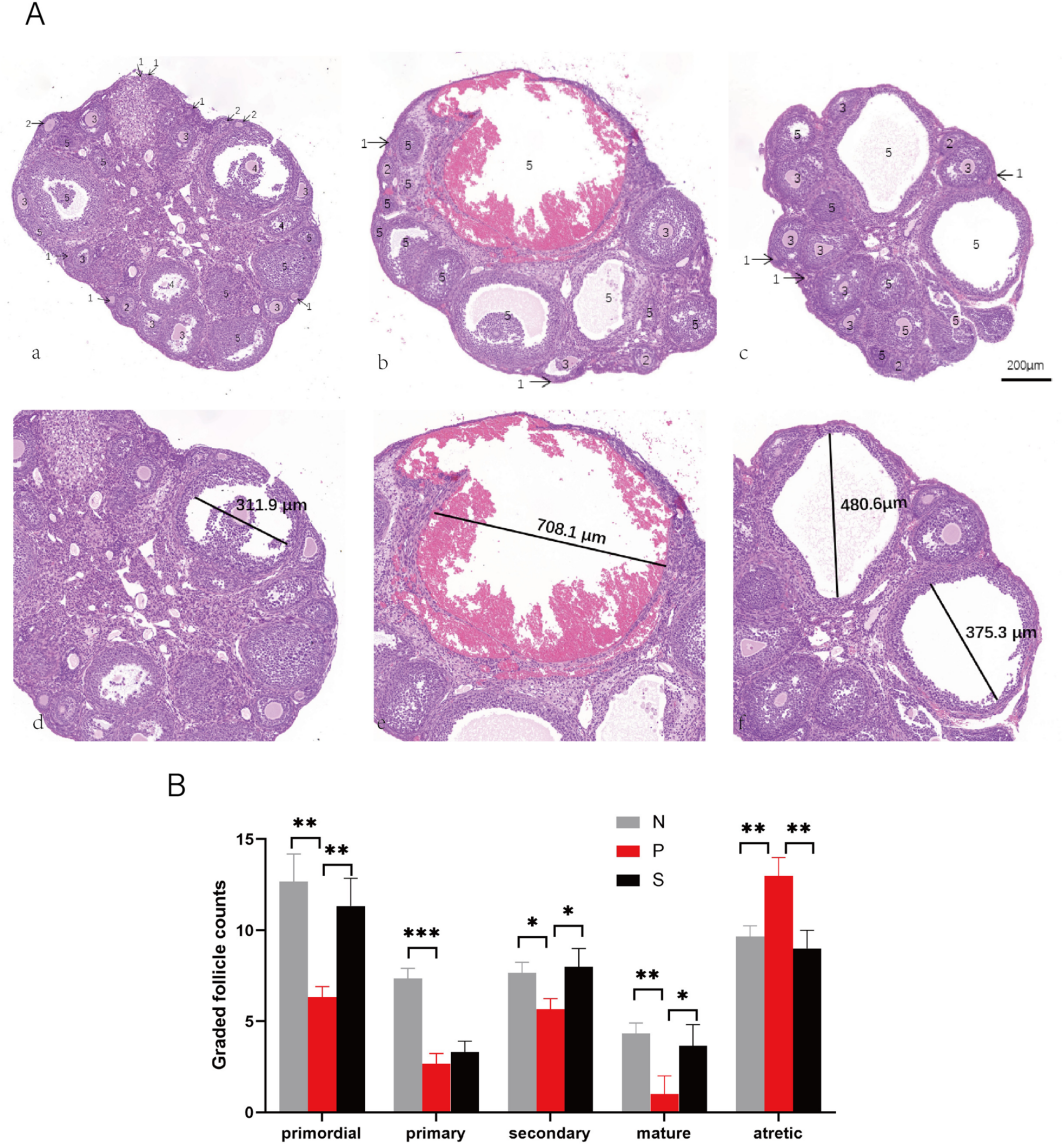
Effect of trehalose on ovarian structure and number of follicles at all levels in mice. **A** HE stained smears of mouse ovaries in each group, a&d: Control group; b&e: PCOS group; c&f: trehalose group (a-c = 5 × microscope; d-f = 10 × microscope); **B** Statistical plot of the number of fifth-order ovarian follicles in each group of mice. N stands for control, P for PCOS, and S for trehalose. Data are presented as M + SD, N group compared with P group, P group compared with S group, *P < 0.05, **P < 0.01, ***P < 0.001, ****P < 0.0001

Normal ovarian tissue is a pair of oval parenchymal organs divided into the medulla and cortex. The medulla is mainly distributed in blood vessels, lymphatic vessels, and nerves. The cortex is thick relative to the medulla. It contains follicles at different stages of development, divided into five stages: primordial follicles (Fig. 3A1), primary follicles (Fig. 3A2), secondary follicles (Fig. 3A3), antral follicles (mature follicles) (Fig. 3A4), and atretic follicles (Fig. 3A5). It can be seen from the figure that the ovarian volume of the PCOS group is larger than that of the Control group, mainly because there are many large cystic follicles (Fig. 3A). There is a difference in the number of follicles at all levels between the PCOS group and the normal group (*P* < 0.05), mainly reflected in the reduction of primordial, primary, secondary, and mature follicles. Among them, primordial follicles decreased more significantly, while the number of atretic follicles increased (Fig. 3B) (*P* < 0.05). After trehalose treatment, the ovarian morphology had good changes; the diameter of ovarian cystic follicles was significantly smaller than that of the PCOS group, the ovarian volume was relatively smaller, and the ovarian follicles at all levels were also improved (*P* < 0.05), mainly in the increase of secondary follicles and the decrease of atretic follicles (*P* < 0.05). However, the number of primary follicles did not change (*P* > 0.05). It proves that the experimental model is successful from the morphological level and provides evidence for trehalose to improve the ovarian function of PCOS.

### Changes in oxidative stress and apoptosis in the ovary

**Fig. 4:**
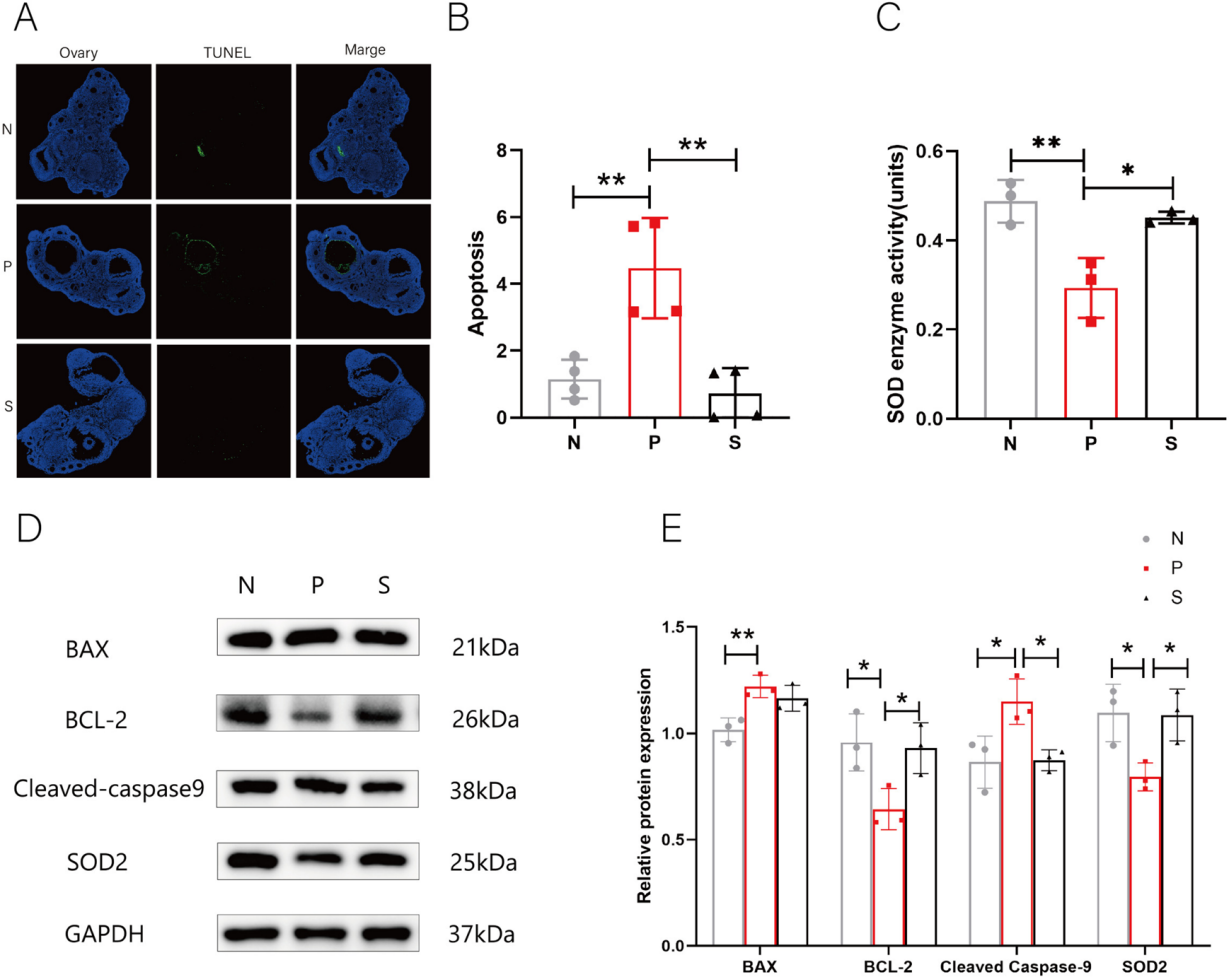
Effect of trehalose on oxidative stress and apoptosis in mouse ovarian mitochondria. **A** Ovarian Tunnel staining of mice in each group; **B** statistical diagram of ovarian Tunnel apoptosis rate in each group; **C** statistical diagram of total SOD content in the ovarian homogenate of mice in each group; **D** Protein bands of BAX, BCL-2, Cleaved caspase-9, SOD2, and GAPDH in ovaries of mice in each group. Each individual band is cropped and distinguished by a black box. The samples were from the same experiment and the gels were processed in parallel. **E** Statistical plots of relative amounts of BAX, BCL-2, Cleaved caspase-9, and SOD2 proteins in the ovaries of mice in each group, and the calculation equation was the gray value of target protein /GAPDH gray value. N stands for control, P for PCOS, and S for trehalose. Data are presented as M + SD, N group compared with P group, P group compared with S group, *P < 0.05, **P < 0.01, ***P < 0.001, ****P < 0.0001

In recent years, PCOS has been associated with mitochondrial damage. The main mechanism is that increased mitochondrial oxidative stress initiates the mitochondrial apoptosis pathway. To investigate the level of oxidative stress, we measured the level of total SOD in ovarian tissue homogenate. We found that the level of total SOD in the ovary was decreased in the PCOS group and increased after trehalose treatment (*P* < 0.05), which was related to the SOD antioxidant family (Fig. 4C), so SOD2 molecules present in mitochondria were selected for detection. WB experiments showed that SOD2 molecules were indeed decreased in the PCOS group and increased after trehalose administration (Fig. 4D-E) (*P* < 0.05). At the same time, the Tunnel test showed that the apoptosis rate (green/blue ratio, blue represents normal cells, green represents apoptotic cells) of PCOS ovaries increased.

In contrast, the apoptosis rate decreased after trehalose treatment (Fig. 4A-B) (*P* < 0.01). To investigate mitochondrial apoptosis, Cleaved caspase-9, an important initiator of the mitochondrial apoptosis pathway, and BAX and BCI-2, which are also known to promote the mitochondrial apoptosis pathway, were selected for WB analysis. The results showed that two apoptotic molecules, BAX and Cleaved caspase-9, were increased, and the BCI-2 anti-apoptotic molecule was decreased in the PCOS group (*P* < 0.05). At the same time, all of them except BAX were improved in the trehalose group (Fig. 4D-E). These alterations indicate that trehalose can ameliorate oxidative stress and apoptosis in the ovaries of PCOS mice.

### Changes in key RAS molecules in ovarian tissue

**Fig. 5:**
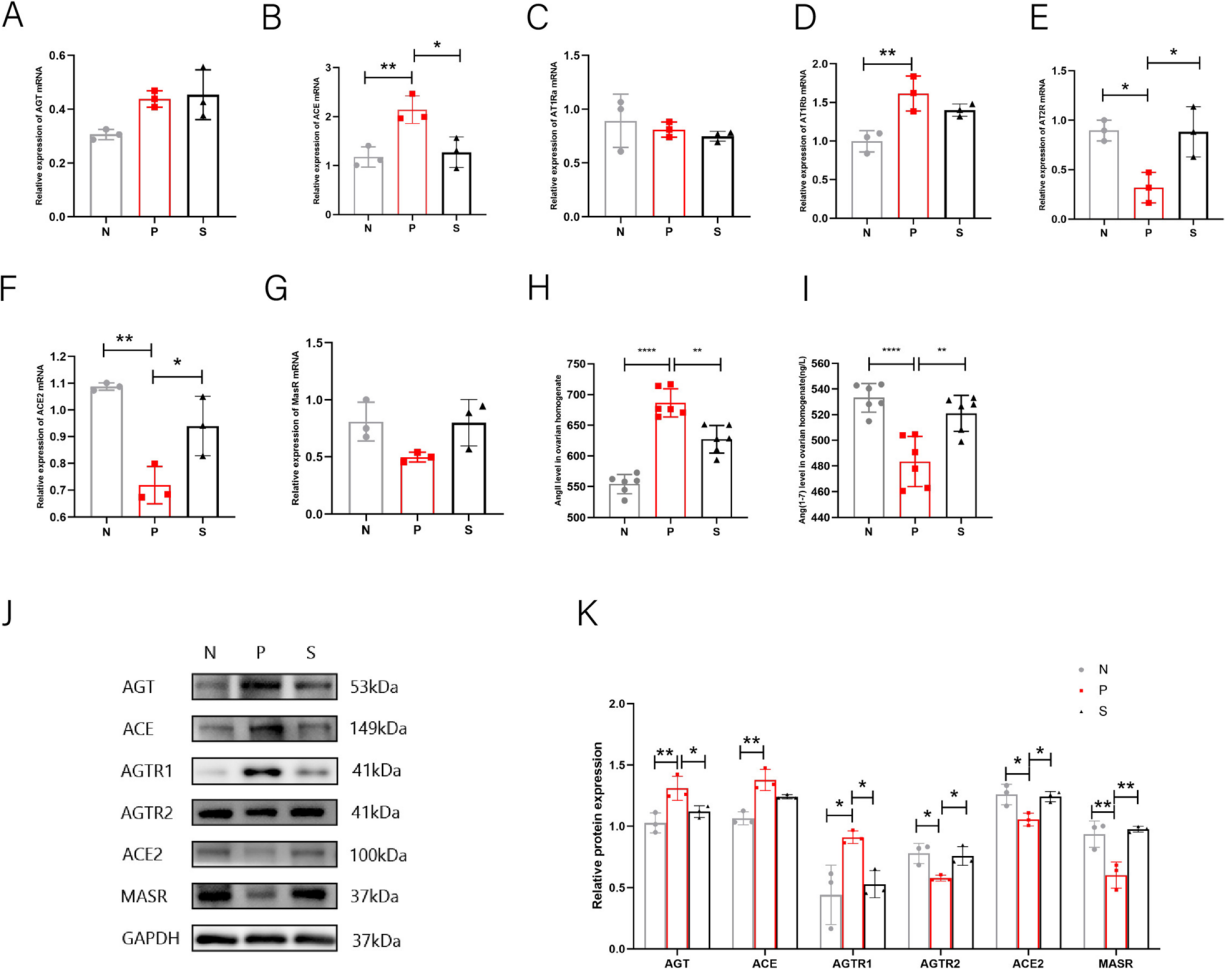
Effect of trehalose on ovarian RAS expression in each group of mice. **A** Ovarian AGT mRNA expression in each group; **B** ovarian ACE mRNA expression; **C** ovarian AT1Ra mRNA expression in each group; **D** ovarian AT1Rb mRNA expression in each group; **E** ovarian AT2R mRNA expression in each group; **F** ovarian ACE2 mRNA expression in each group; **G** expression of ovarian MASR mRNA in each group; **H** ovarian AngII protein content in each group; **I** ovarian Ang1-7 protein content; **J** protein band diagram of ovarian RAS in each group. Each individual band is cropped and distinguished by a black box. The samples were from the same experiment and the gels were processed in parallel; **K** Statistical plots of relative protein expression of RAS in mouse ovaries of each group. N stands for control, P for PCOS, and S for trehalose. Data are presented as M + SD, N group compared with P group, P group compared with S group, *P < 0.05, **P < 0.01, ***P < 0.001, ****P < 0.0001

OVRAS are known to affect ovarian function and are associated with ovarian steroidogenesis, follicle maturation, and atresia, and ACE genotypes are strongly associated with PCOS characteristics in PCOS studies. In recent years, researchers have found that many diseases are mainly caused by the imbalance of the two classical pathways in RAS, but no one has proposed whether the two pathways are unbalanced in PCOS. Our study mainly conducted quantitative analysis at the molecular level of mRNA and protein and found that ACE/AngII/AT1R was increased, ACE2/Ang1-7/MASR and AT2R were decreased in PCOS (Fig. 5) (*P* < 0.05), which confirmed the imbalance of the two pathways in PCOS for the first time. In the future, we can block the ACE/AngII/AT1R pathway and activate ACE2/Ang1-7/MASR and AT2R to treat PCOS. We also found that trehalose changed the trend of the two pathways while improving the symptoms of PCOS (*P* < 0.05). In the mRNA expression of AGT, AT1R and MASR did not change, ACE was down-regulated, while ACE2 and AT2R were up-regulated. At the protein level, AGT, AngII, and AT1R were up-regulated, ACE2, Ang1-7, MASR, and AT2R were down-regulated, while ACE did not change. Our results suggest that two classical pathways in OVRAS are involved in the pathogenesis of PCOS and that trehalose exerts its therapeutic effect through OVRAS.

### Trehalose ameliorated oxidative stress and apoptosis in DHEA-treated KGN cells

**Fig. 6:**
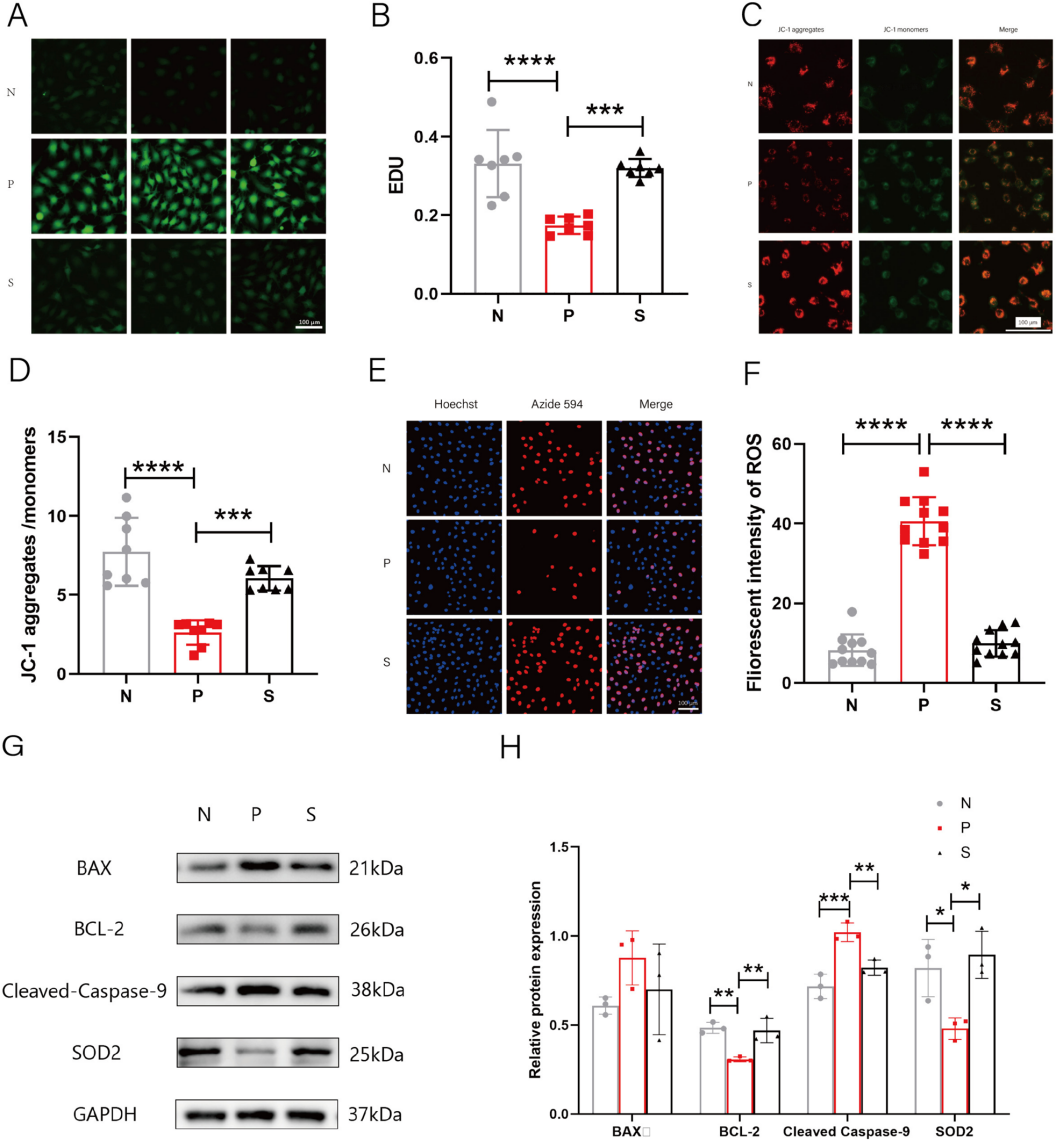
Effects of trehalose on intracellular oxidative stress and apoptosis and proliferation of KGN cells in each group. **A** ROS immunofluorescence staining of DCFH-DA probe in each group; **B** Statistical plot of differences in ROS levels of DCFH-DA probes in each group; **C** The fluorescence of JC-1 aggregate (red light), JC-1 monomer (green light), and combined fluorescence (red/green light); **D** Statistical plot of (JC-1 aggregate fluorescence intensity)/(JC-1 monomer fluorescence intensity) ratio in each group; **E** Hoechst(blue light), Azide 594(red light), and their combined light (red and blue light); **F** Numerical statistics of (Azide 594 red light spot number)/(Hoechst blue light spot number) in each group; **G** Protein bands of BAX, BCL-2, Cleaved caspase-9, SOD2, and GAPDH in KGN cells of each group; Each individual band is cropped and distinguished by a black box. The samples were from the same experiment and the gels were processed in parallel; **H** Statistical plots of relative amounts of BAX, BCL-2, Cleaved caspase-9, and SOD2 proteins in KGN cells of each group, were calculated by the gray value of target protein /GAPDH gray value. N stands for control, P for PCOS, and S for trehalose. Data are presented as M + SD, N group compared with P group, P group compared with S group, *P < 0.05, **P < 0.01, ***P < 0.001, ****P < 0.0001

Cells (a human granulosa cell tumor cell line) to investigate the effect of trehalose on PCOS. “Based on our previous KGN model, in which cells were co-cultured with 10-5DHEA for 48 h, we mentioned that oxidative stress in mitochondria in PCOS ovaries could trigger apoptosis, but we did not know whether similar results would be observed in granulosa cells”. As expected, we found an increase in the level of oxidative stress ROS, which was significantly decreased after trehalose treatment (Fig. 6A-B) (*P* < 0.0001).

Mitochondria are essential organelles in energy metabolism, accounting for 95% of the total ATP output in humans. A precursor to normal mitochondrial function is the stabilization of mitochondrial membrane potential, and a decrease in mitochondrial membrane potential can predict early apoptosis. In this experiment, the mitochondrial membrane potential of group P (PCOS group) was decreased (*P* < 0.0001), while after trehalose treatment, the mitochondrial membrane potential was significantly increased (Fig. 6C-D) (*P* < 0.0001). Apoptosis and proliferation are closely coordinated in the organism, and the balance between them is crucial for maintaining life activities. To detect the proliferation of KGN cells, we chose a more sensitive EDU experimental method. EdU can be incorporated into the newly synthesized DNA instead of thymidine during DNA synthesis, and the proliferating cells show very bright red fluorescence under the fluorescence microscope, which can better detect the proliferation of cells. We found that the proliferation rate of KGN cells was significantly decreased after DHEA modeling. In contrast, the proliferation rate was significantly increased after trehalose treatment compared with the DHEA group (Fig. 6E-F) (*P* < 0.001).

To better illustrate the anti-oxidation, anti-apoptosis, and pro-proliferation effects of trehalose in KGN cells, we selected the antioxidant molecules SOD2, pro-apoptotic molecules BAX, Cleaved caspase-9 molecules and anti-apoptotic molecules BCL-2 that are altered in PCOS mouse ovaries. We performed WB experiments and found that cleaved caspase-9 was increased, and SOD2 and BCL-2 were decreased in the KGN model. After trehalose treatment, Cleaved caspase-9 was decreased, SOD2 and BCL-2 were increased (*P* < 0.05), and BAX expression was always not changed (Fig. 6G-H) (*P* > 0.05).

Our experiments illustrate that trehalose can ameliorate oxidative stress and reduce apoptosis in PCOS at both phenotypic and molecular levels.

### Changes in key RAS components in KGN cells

**Fig. 7:**
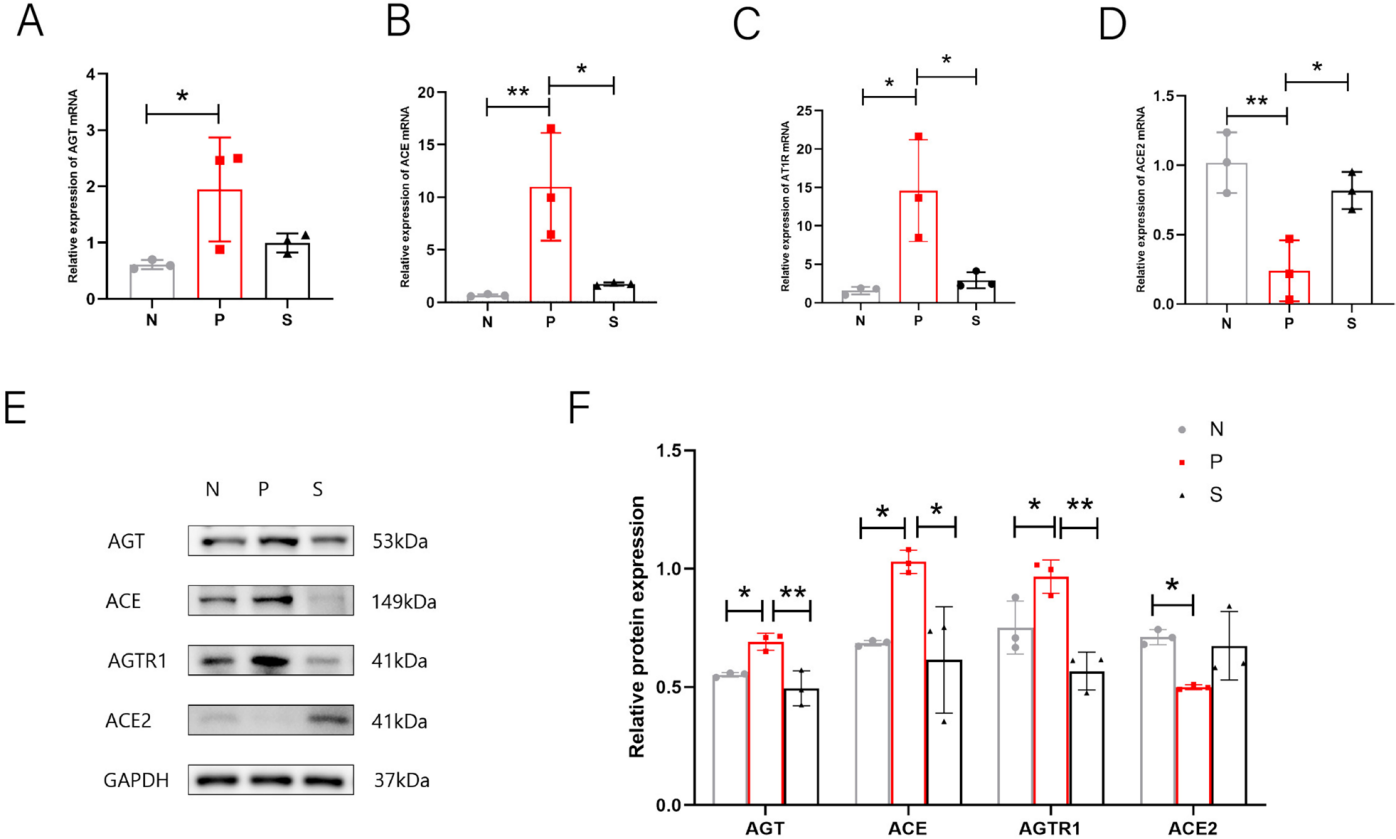
Effect of trehalose on RAS expression in KGN cells of each group. **A** AGT mRNA expression in KGN cells of each group; **B** ACE mRNA expression in KGN cells; **C** AT1R mRNA expression in KGN cells of each group; **D** ovarian ACE2 mRNA expression in each group; **E** RAS protein band pattern of KGN cells in each group; Each individual band is cropped and distinguished by a black box. The samples were from the same experiment and the gels were processed in parallel; **F** Statistical plot of relative protein expression of RAS in KGN cells of each group. N stands for control, P for PCOS, and S for trehalose. Data are presented as M + SD, N group compared with P group, P group compared with S group, *P < 0.05, **P < 0.01, ***P < 0.001, ****P < 0.0001

We further explored whether the molecular mechanism of RAS in granulosa cells in PCOS differs from that in the ovary, and again, we selected the mRNA and protein expression assays of two classical Ras pathway molecules. Our study found that KGN cells do not express AT2R and MASR, neither at the mRNA or protein levels. We found that AGT, ACE, and AT1R increased significantly in DHEA, while ACE2 decreased. After trehalosaccharine treatment, ACE, and AT1R decreased in mRNA and protein levels, while AGT and ACE2 were different in mRNA and protein levels. The mRNA expression of AGT and ACE2 remained unchanged/increased. However, the protein level was decreased/unchanged, and the expression of ACE2 was the lowest (Fig. 7) (*P* < 0.05), indicating that there may be experimental error. We look forward to using more samples to explain this phenomenon. In general, the molecular changes of KGN in PCOS are consistent with those in the ovary, and the changes of trehalose in KGN cells (DHEA model) mainly focus on the AT1R molecule, which is the receptor for our future study.

## Discussion

The exact etiology of PCOS is still unclear, and it is mainly believed to be the combined effect of genetics and living environment. With the rapid development of modern society, people’s lives and psychological pressure are increasing, and the number of PCOS patients is generally greatly increased; most symptoms appear in adolescence and seriously affect the fertility needs of women of childbearing age, to a large extent, damage the physical and mental health of women’s lifetime [41]. Because of its complex etiology and high heterogeneity, it is impossible to explain its root cause. There are no specific drugs or fixed means to control the disease. At present, hormone therapy is mainly used to meet the needs of patients, but hormones will bring many adverse effects. Therefore, many researchers have found that some vitamins, minerals, and some specific drugs (such as curcumin [42], etc.) have therapeutic effects on PCOS, and patients tend to use such nutritional supplements without side effects on the body [43]. It is undoubtedly a new and bright way for the treatment of PCOS.

In recent years, trehalose has attracted much attention because of its ability to help many diseases (degenerative diseases, etc.), and it is structurally stable, easy to manufacture, and affordable. It does not need to be tested for use. Because trehalose can also reduce blood glucose and insulin resistance [26], we wonder whether it will also be helpful for the treatment of PCOS. Our study shows that trehalose is also beneficial for PCOS and has been completely non-harmful in the human body for a long time. Our experiments show that trehalose can improve the body weight, polycystic ovary, and follicle number at all levels (by decreasing the number of atretic follicles, and increasing the number of primordial follicles, secondary follicles, and mature follicles), glucose tolerance Insulin resistance and T, E2, INS, and AMH hormone levels in PCOS mice. At the same time, trehalose can also improve oxidative stress and apoptosis in PCOS mice ovaries and KGN. Here, we mainly study the effect of trehalose through OVRAS. We will add a new option and further mechanism for future nutritional supplements for PCOS.

In recent years, researchers have found that mitochondrial damage may cause PCOS. Researchers have found that the production of cellular mitochondrial ROS is related to insulin resistance and its complications, and mitochondrial gene mutations are often associated with PCOS women [44]. It may be due to the decrease of antioxidant enzymes, leading to the massive production of mitochondrial ROS [45], triggering the mitochondrial apoptotic pathway and induced cell apoptosis. SOD2, which is mainly found in mitochondria, has an antioxidant effect. With decreased mitochondrial membrane potential and decreased mitochondrial membrane permeability, cytochrome C released from mitochondria to cytoplasm cleaved caspase-9 to form cleaved-caspase-9, which cleaved caspase-3. The formed cleaved-caspase-3 cleaves PARP, leading cells to apoptosis. The increase of the pro-apoptotic protein BAX and the decrease of the anti-apoptotic protein Bcl2 can also promote the decrease of mitochondrial membrane potential and cell apoptosis. Our experiments demonstrated that PCOS mice had increased ovarian apoptosis and decreased total SOD activity in ovarian homogenate, as well as decreased mitochondrial membrane potential, increased ROS, and decreased edu-stained cell proliferation rate in KGN cells after modeling, all of which were improved after trehalose treatment.

Moreover, in the tissues and cells of the PCOS model group, it was found that SOD2 antioxidant molecules showed a downward trend, and after trehalose treatment, it showed an upward trend. In contrast, Cleaved caspase-9 showed an upward trend, and BCL-2 anti-apoptotic molecules showed a downward trend. After trehalose treatment, Cleaved caspase-9 was down-regulated, BCL-2 was up-regulated, but BAX was not changed, proving that trehalose could treat PCOS through anti-oxidation and anti-apoptosis.

PCOS is characterized by hyperandrogenism, insulin resistance, and ultrasonographic polycystic ovaries. Because obtaining ovarian tissue from PCOS patients is almost impossible, many researchers have adopted various animal models to simulate PCOS disease. We used the PCOS mouse model of subcutaneous injection of DHEA in the neck and back combined with high-fat feeding used by most researchers [46] and KGN cells with DHEA [47] by observing their changes to explore the mechanism behind human PCOS. RAS is mainly divided into two classical pathways: ACE/AngII/AT1R/AT2R and ACE2/Ang1-7/MasR, but AT1R and AT2R are antagonistic. The molecules of these two pathways have been shown to exist in the human ovary [30, 48], which are also the molecules we mainly explore in this article. Early studies found that RAS is associated with a range of reproductive activities, such as follicular development, steroidogenesis [49, 50], oocyte maturation, ovulation [51], and follicular atresia, and is regulated by gonadotropins. Subsequently, components of OVRAS are closely related to the pathological features of PCOS. The main studied molecules are prorenin, angiotensinogen, and ACE. Researchers found that increased serum total renin in PCOS patients may be related to androgen [52]. The M235T polymorphism of the angiotensinogen gene is related to insulin resistance in PCOS patients, and the genotype of ACE is related to the occurrence and development of PCOS women. The specific genotype differences vary from region to region [53], and the angiotensin-converting enzyme inhibitor lisinopril can reduce serum androgen levels in PCOS patients [54]. However, the other classical RAS molecules have not been elucidated and need further study. Therefore, we further performed a supplementary experiment with PCOS mice. Our study found that ACE/AngII/AT1R increased and ACE2/Ang1-7/MasR and AT2R decreased in the ovaries of PCOS mice.

Since previous studies have shown strong immunostaining for renin and angiotensin in the theca cells and granulosa cells (GC) surrounding large cystic follicles in PCOS patients, while in normal ovarian follicular development, immunostaining is limited to the theca cell layer, except for follicles immediately before ovulation, which show strong staining for granulosa and theca cells [55], We hypothesized that RAS in granulosa cells may be responsible for promoting the overdevelopment of follicles in PCOS patients, resulting in the cystic appearance of follicles. Therefore, we further studied to explore the changes of RAS in granulosa cells in PCOS and to find out the cause of oocyte dysfunction in PCOS patients. Because it is difficult to obtain granulosa cells from PCOS patients, we used the KGN cell model, which is more used in PCOS cell models [56]. In the KGN cell model, the PCOS group showed an upward trend of AGT, ACE, and AT1R, and a downward trend of ACE2. The expression of AT2R and MASR was not detected. According to the previous literature, the expression of MAS in granulosa cells was verified by immunohistochemistry next to follicular cells at all levels in normal women, and human granulosa cells also expressed AT2R [57]. However, we did not find the mRNA and WB expression of MAS and AT2R in KGN cells. The KGN cell line may not fully represent normal human granulosa cells in these two molecules, which is a defect of this paper. In the future, granulosa cells from PCOS patients may be used for further study. Some studies have shown that granulosa cells in PCOS patients highly express AT1R and AT2R, However, the expression of AT2R is far less than that of AT1R, and it is found that the expression of AT1R is negatively correlated with the amount of FSH used to induce ovulation [57]. Our findings are consistent with previous studies and suggest that the ACE/AngII/AT1R pathway is highly expressed in the KGN cell model of PCOS, suggesting that the ACE/AngII/AT1R pathway may promote excessive follicle development and hinder ovulation in PCOS patients. Our findings provide some complement to previous studies. It provides a further mechanism for PCOS disease, and better elaborates the correlation and changing trend of OVRAS in PCOS disease.

After treatment with trehalose, the phenotypes of PCOS mice and KGN cells were improved. In molecular mechanism, the expression of ACE/AngII/AT1R decreased, and ACE2/Ang1-7/MasR and AT2R increased in PCOS mice ovaries, while AGT, ACE, and AT1R decreased in KGN cells. However, it did not change the expression of ACE2. We propose for the first time that the two classical pathways of OVRAS are closely related to the occurrence and development of PCOS, in which ACE/AngII/AT1R can promote the deterioration of PCOS symptoms. At the same time, ACE2/Ang1-7/MasR and AT2R can improve the symptoms of PCOS. The two pathways are antagonistic, and imbalance will lead to the occurrence and development of PCOS.

The renin-angiotensin system (RAS) has been found by many researchers to be associated with oxidative stress and apoptosis in diseases, in which ACE/AngII/ATR1 promotes oxidative stress and apoptosis [58] and ACE2/Ang1-7/ MasR reduces oxidative stress and apoptosis [59]. However, the relationship between RAS and oxidative stress and apoptosis in ovarian and KGN cells has not been studied. Our data showed that elevated ACE/AngII/ATR1 and decreased ACE2/Ang1-7/MASR were associated with increased oxidative stress and apoptosis, suggesting that RAS is associated with oxidative stress and apoptosis in ovarian and KGN cells.

In conclusion, the present study demonstrated that trehalose could improve ovarian function and insulin resistance in PCOS mice and exerted anti-oxidative and anti-apoptotic effects at both tissue and cellular levels. Our experiments further shed light on the relationship between local RAS and PCOS, and trehalose may act through local RAS.Although the activation/inhibition of specific molecules was not used for causal validation in this study, OVRAS are associated with oxidative stress and apoptosis in other diseases, and we will further investigate by targeting ATR1,ATR2 and MASR in the future. This study verified that the changes of important molecules of OVRAS were related to oxidative stress and apoptosis, which provided experimental and theoretical basis for trehalose to treat PCOS in the future. The importance of OVRAS was further elaborated, which provided ideas and directions for subsequent research.

### Supplementary Information

**Additional file 1.**

## Data Availability

The data sets used and/or analyzed during the current study are available from the corresponding author upon reasonable request.
