# Supplementary material for: Trehalosemodulates OVRAS to improve oxidative stress and apoptosis in KGN cells and ovaries of PCOS mice
Source: J Ovarian Res. 2024 Jan 9;17:11. doi: 10.1186/s13048-023-01337-5 (PMC10775634; doi:10.1186/s13048-023-01337-5)

Supplementary figure1.Western Blot gels of BAX

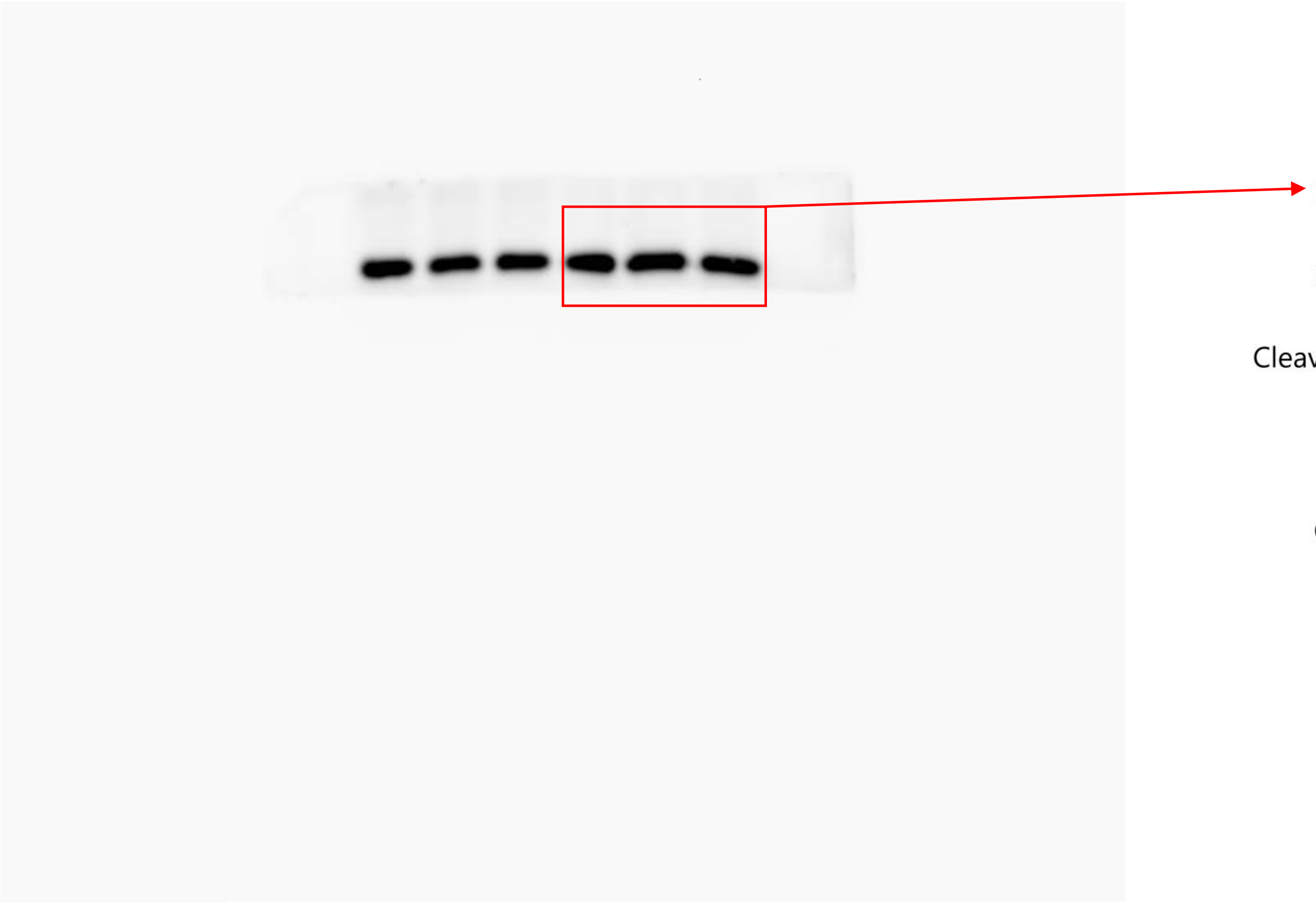

Figure4 （D）

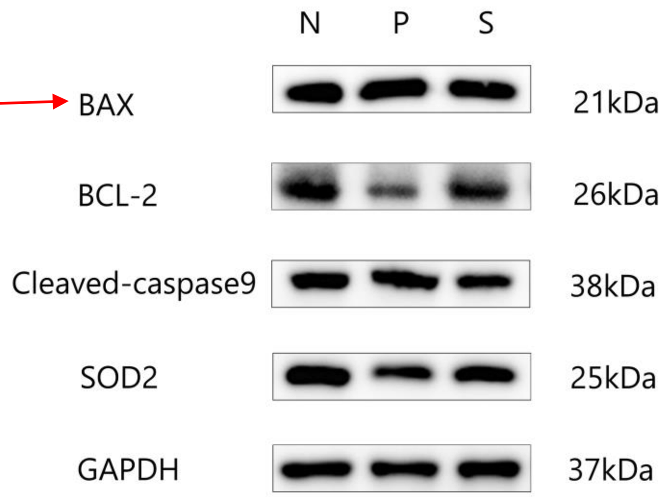

Supplementary figure2.Western Blot gels of BCL-2

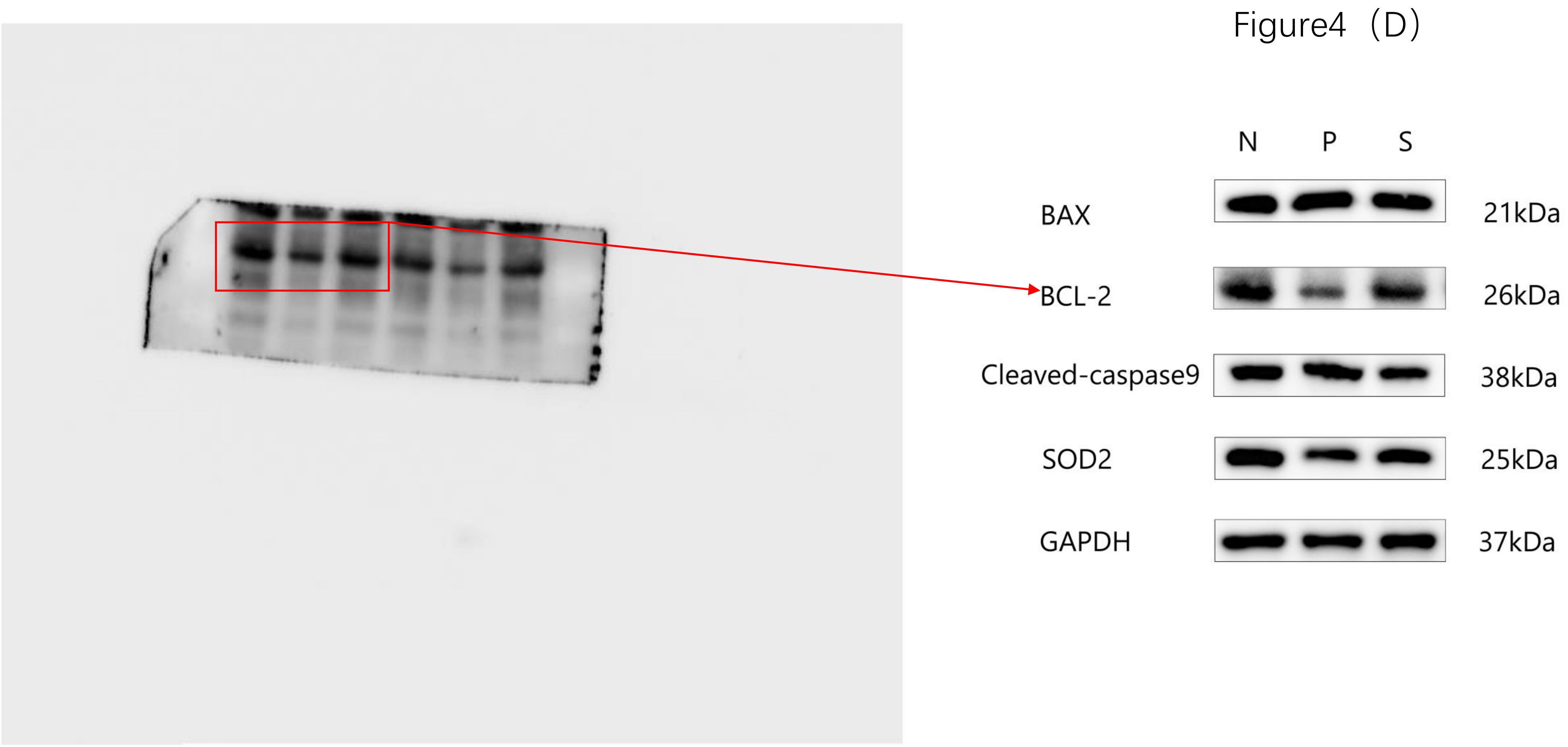

Supplementary figure3.Western Blot gels of Cleaved-Caspase-9

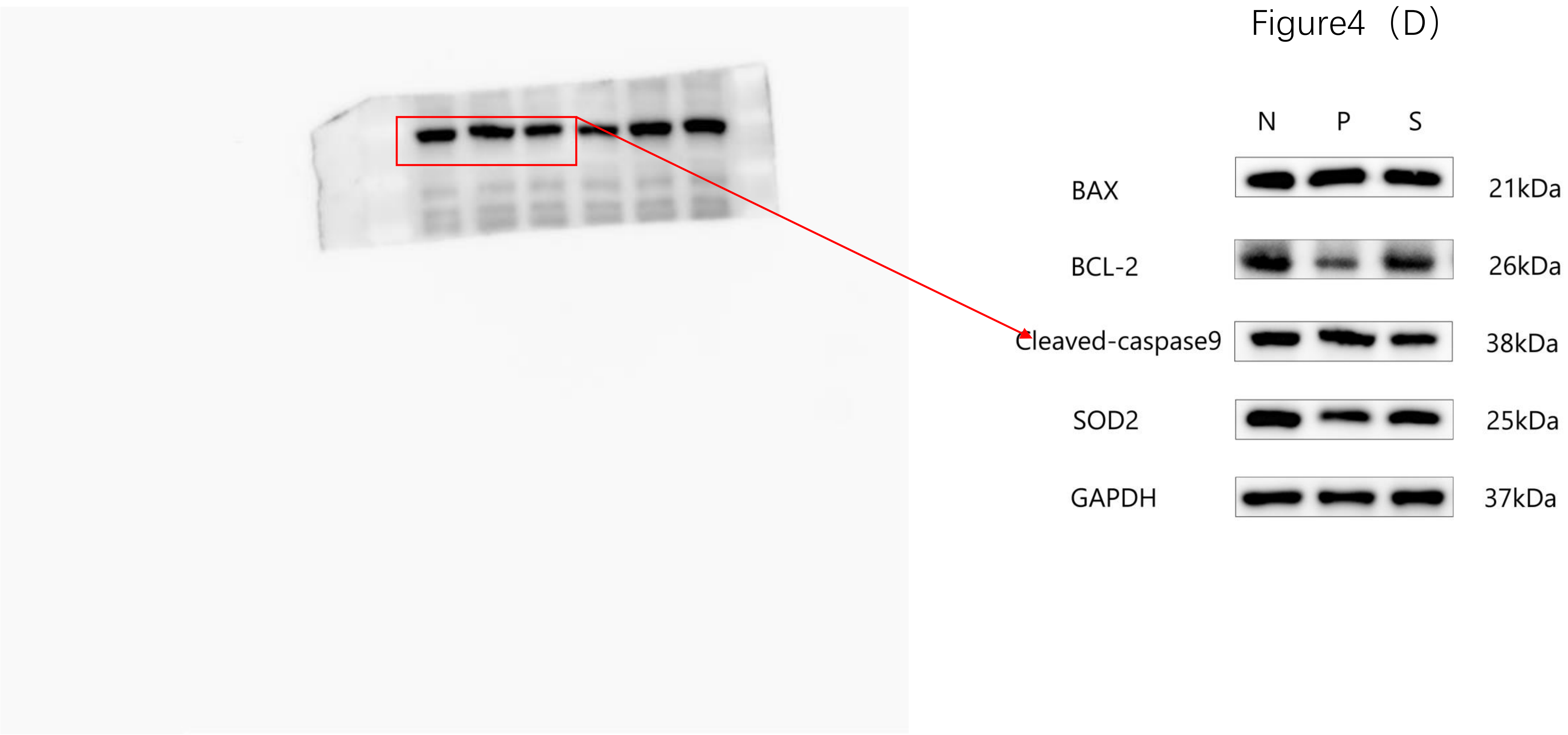

Supplementary figure4.Western Blot gels of SOD2

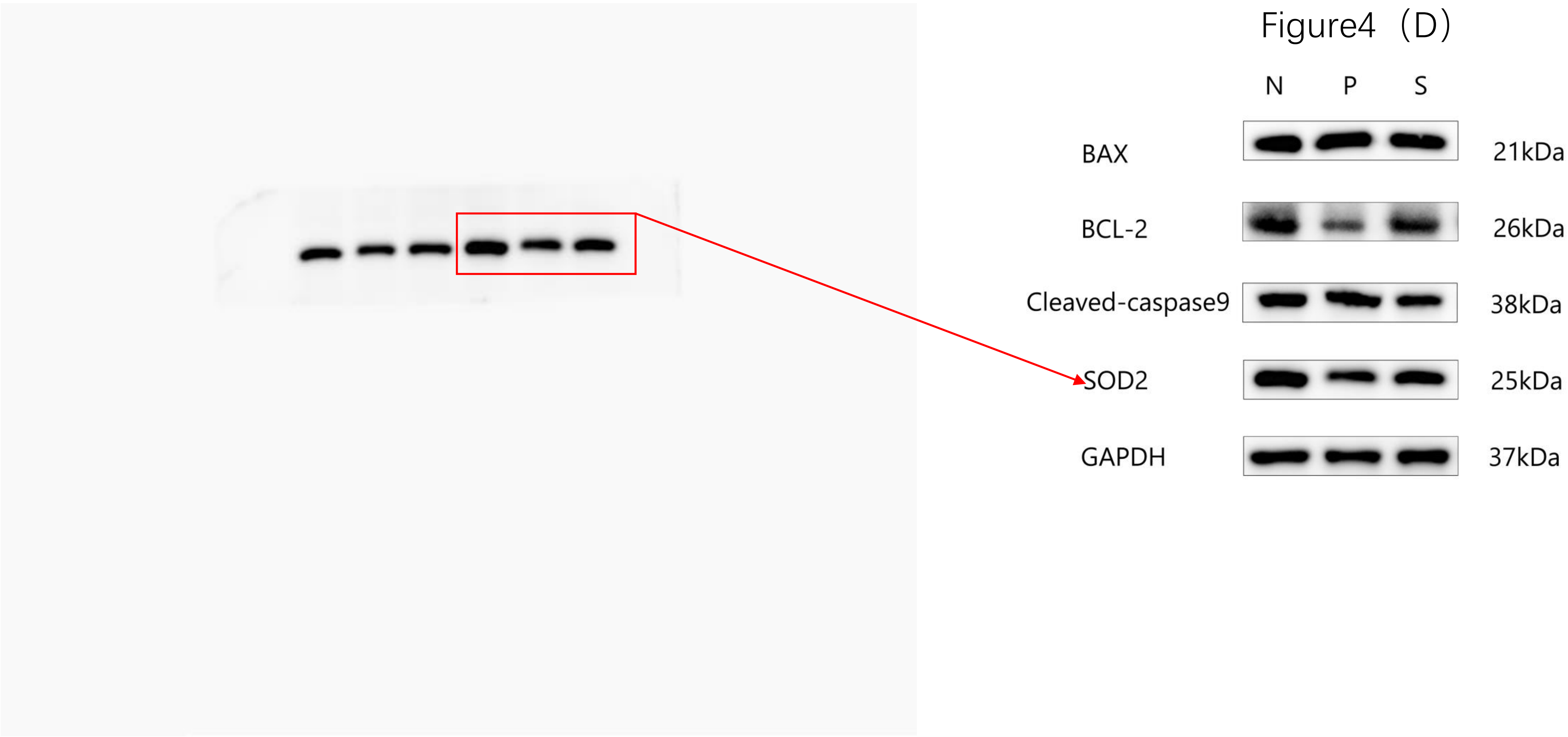

Supplementary figure5.Western Blot gels of GAPDH

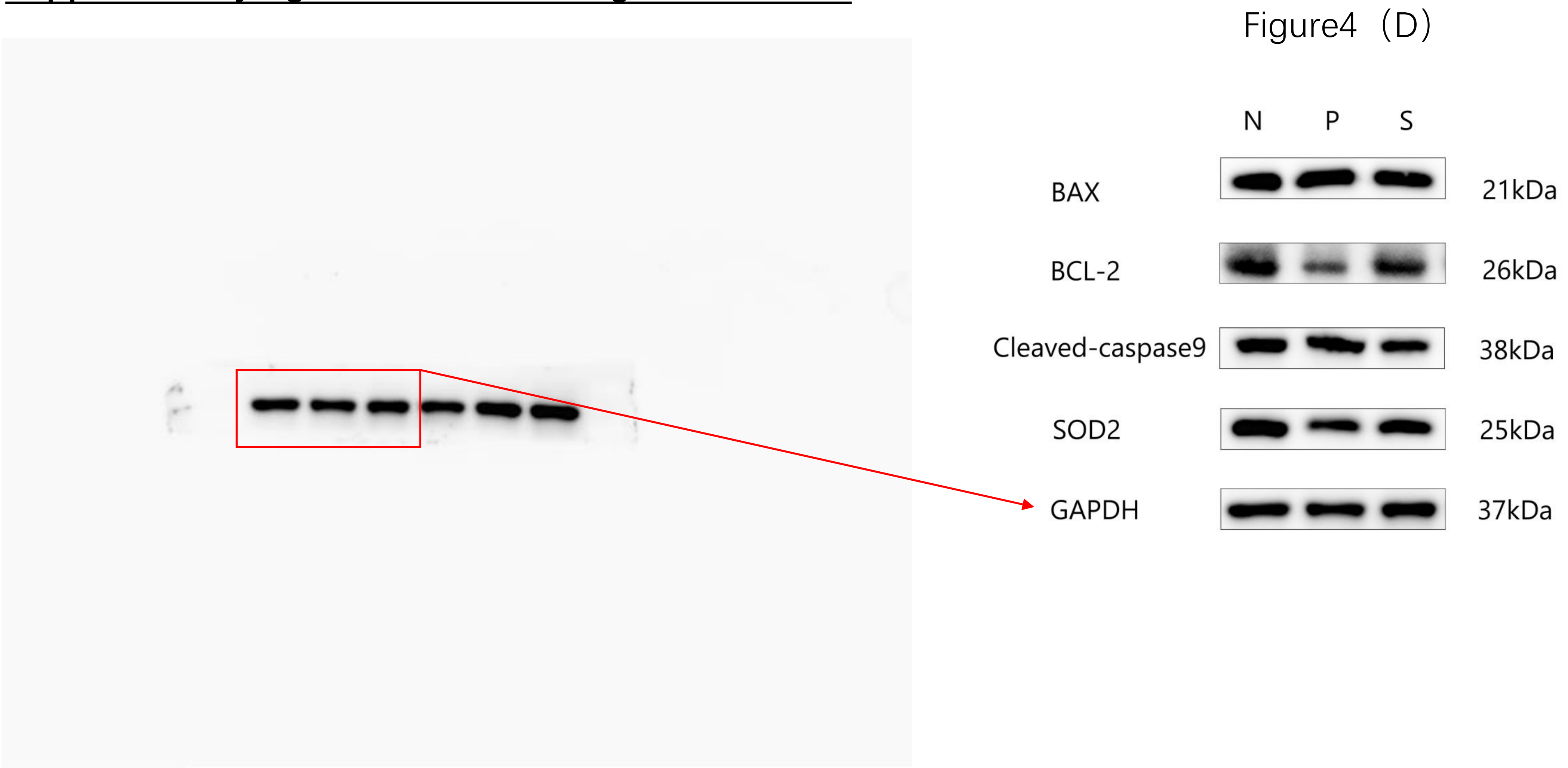

Supplementary figure6.Western Blot gels of AGT

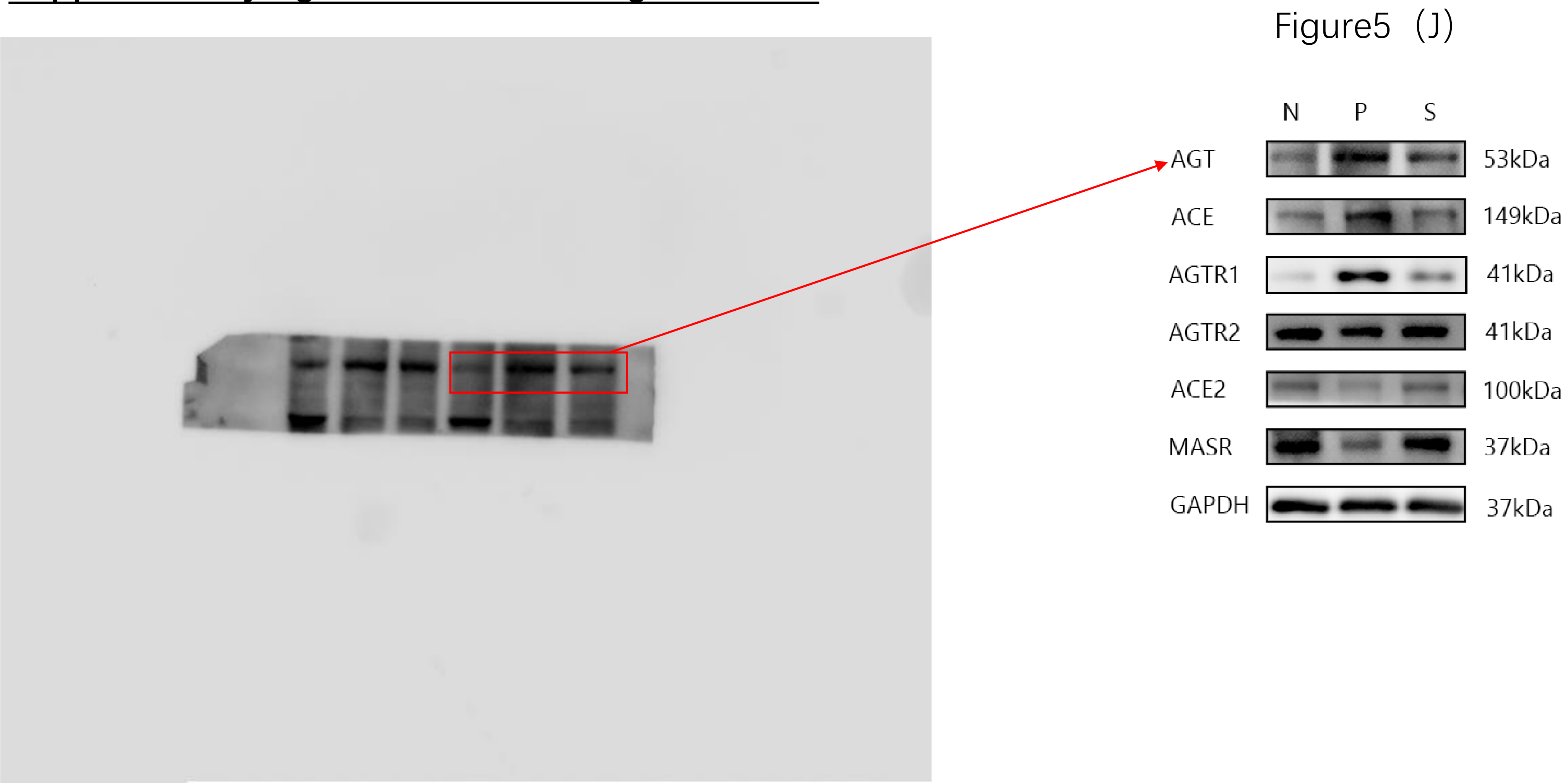

Supplementary figure7.Western Blot gels of ACE

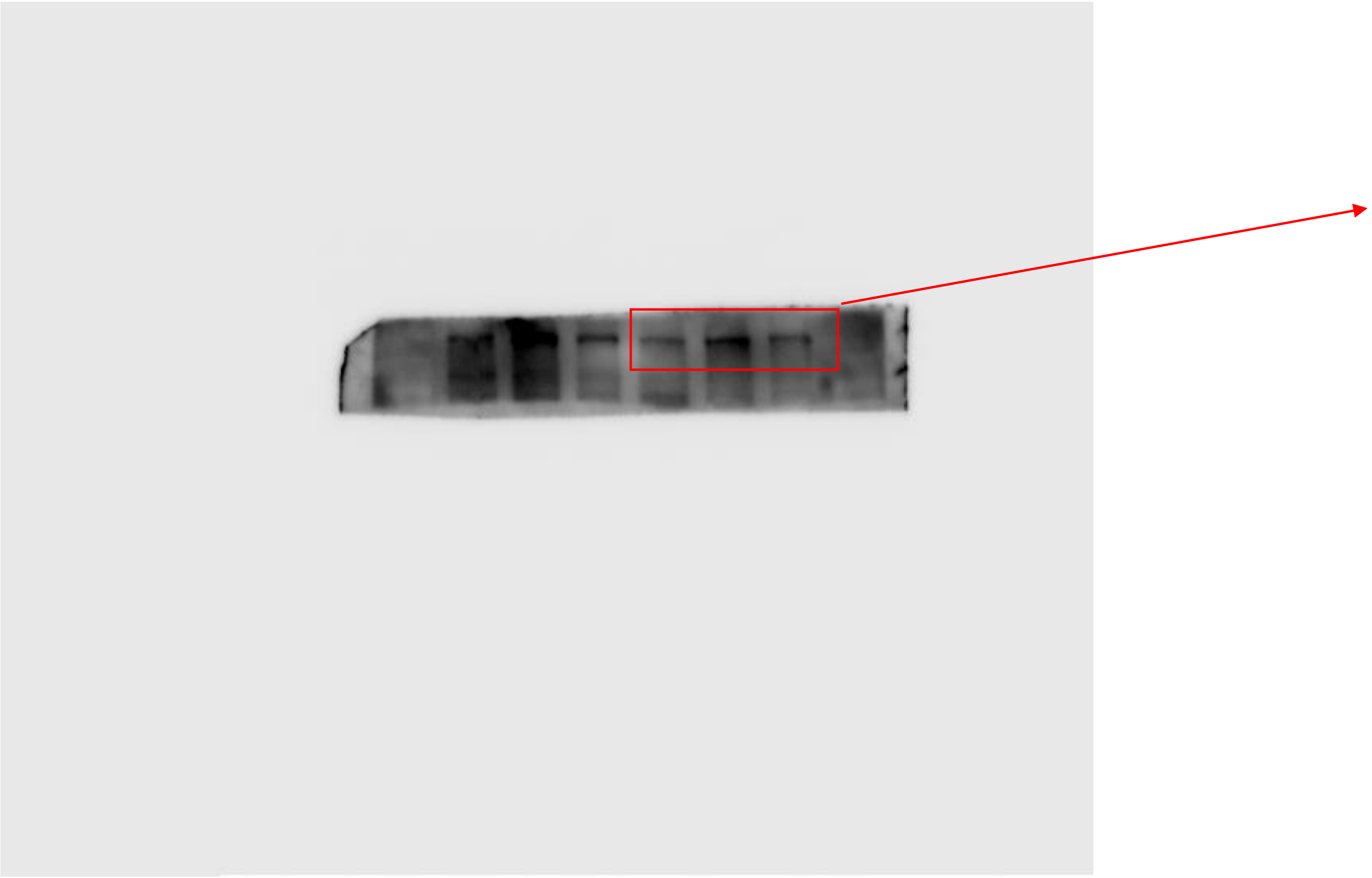

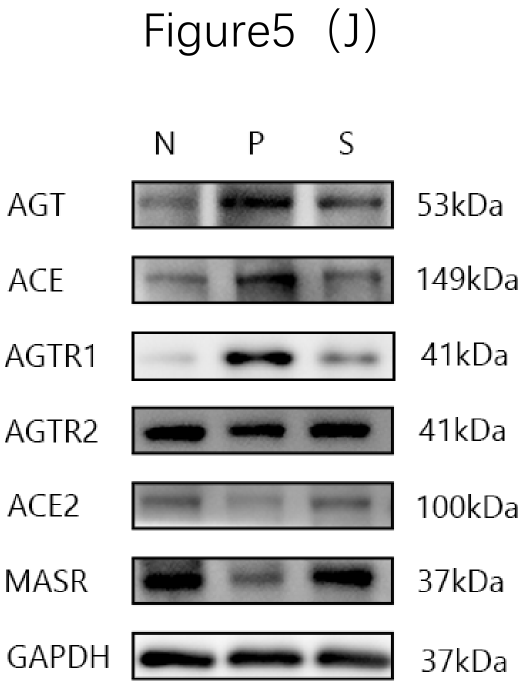

Supplementary figure8.Western Blot gels of AGTR1

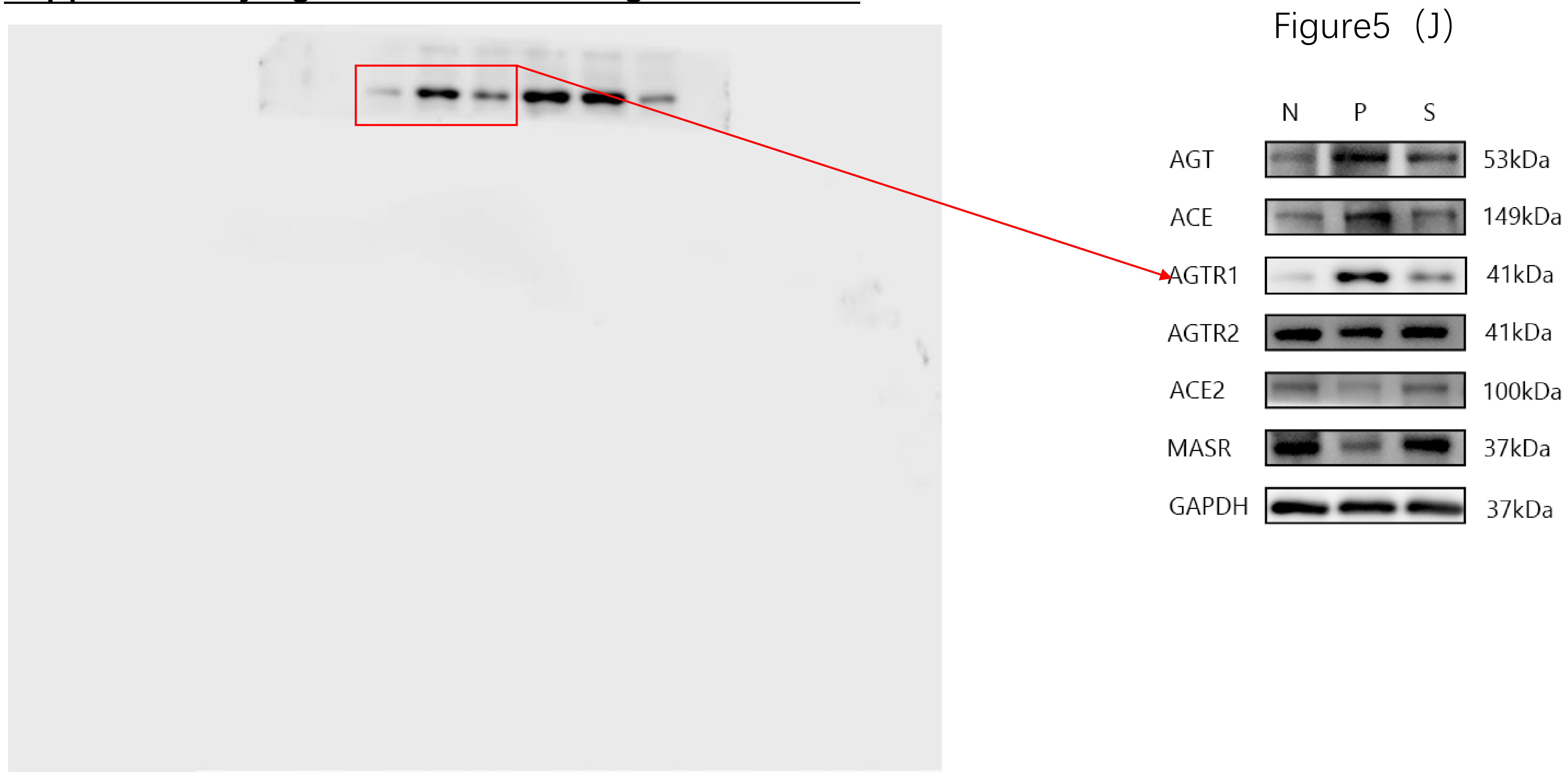

Supplementary figure9.Western Blot gels of AGTR2

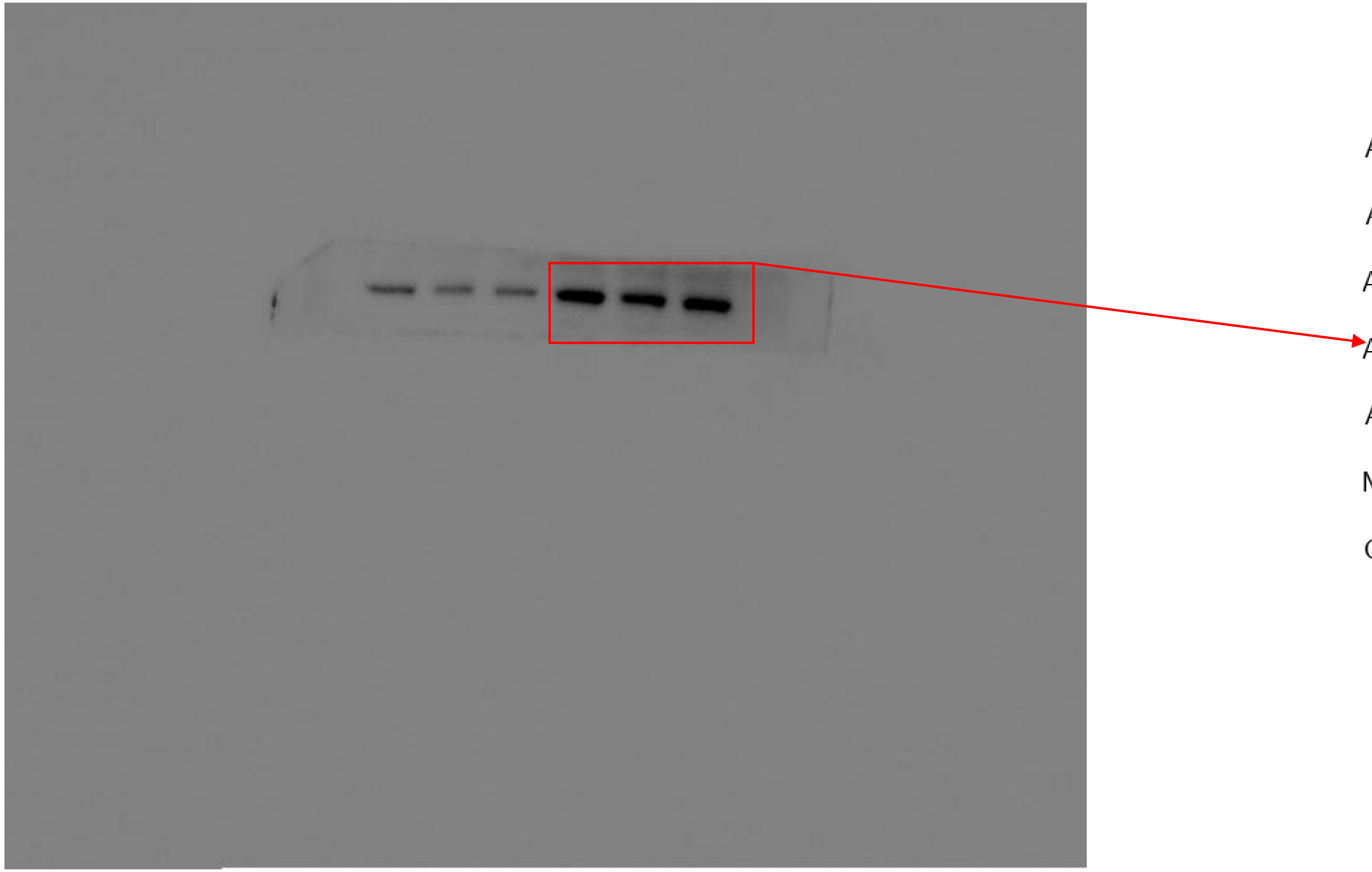

Figure5 （J）

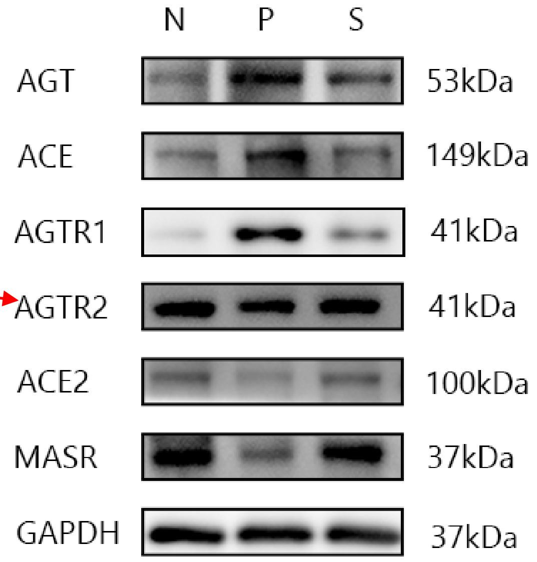

Supplementary figure10.Western Blot gels of ACE2

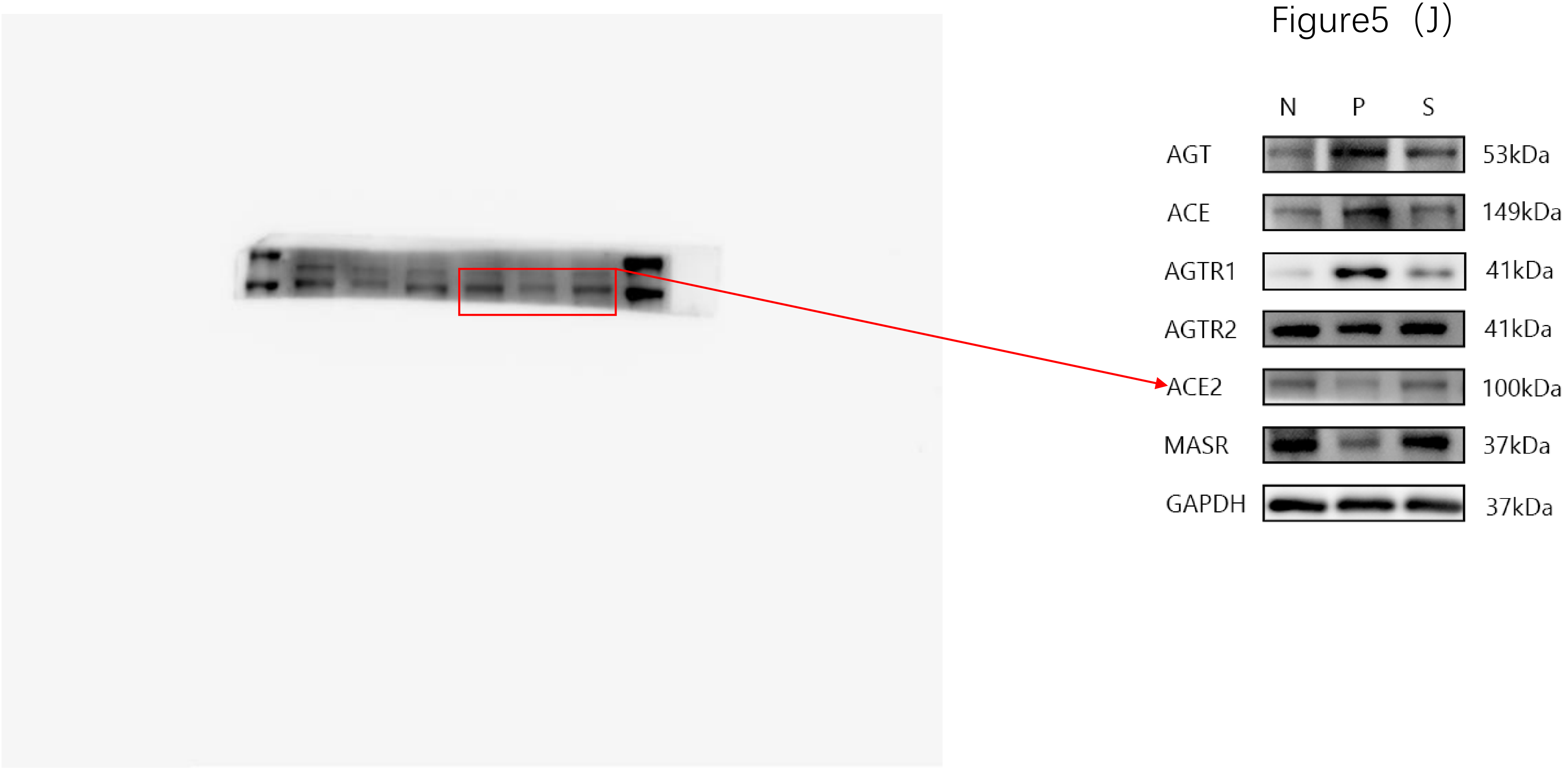

Supplementary figure11.Western Blot gels of MASR

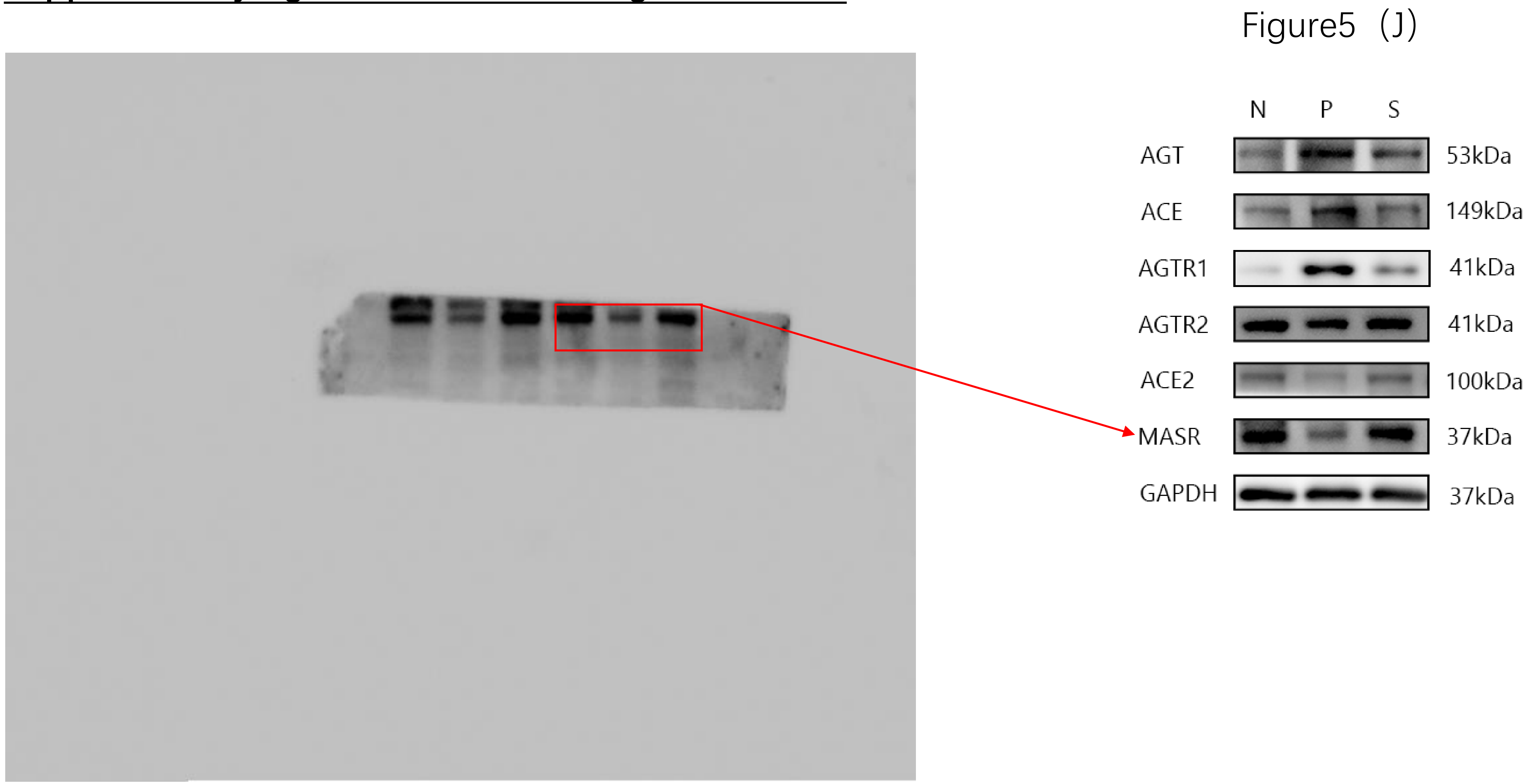

Supplementary figure12.Western Blot gels of GAPDH

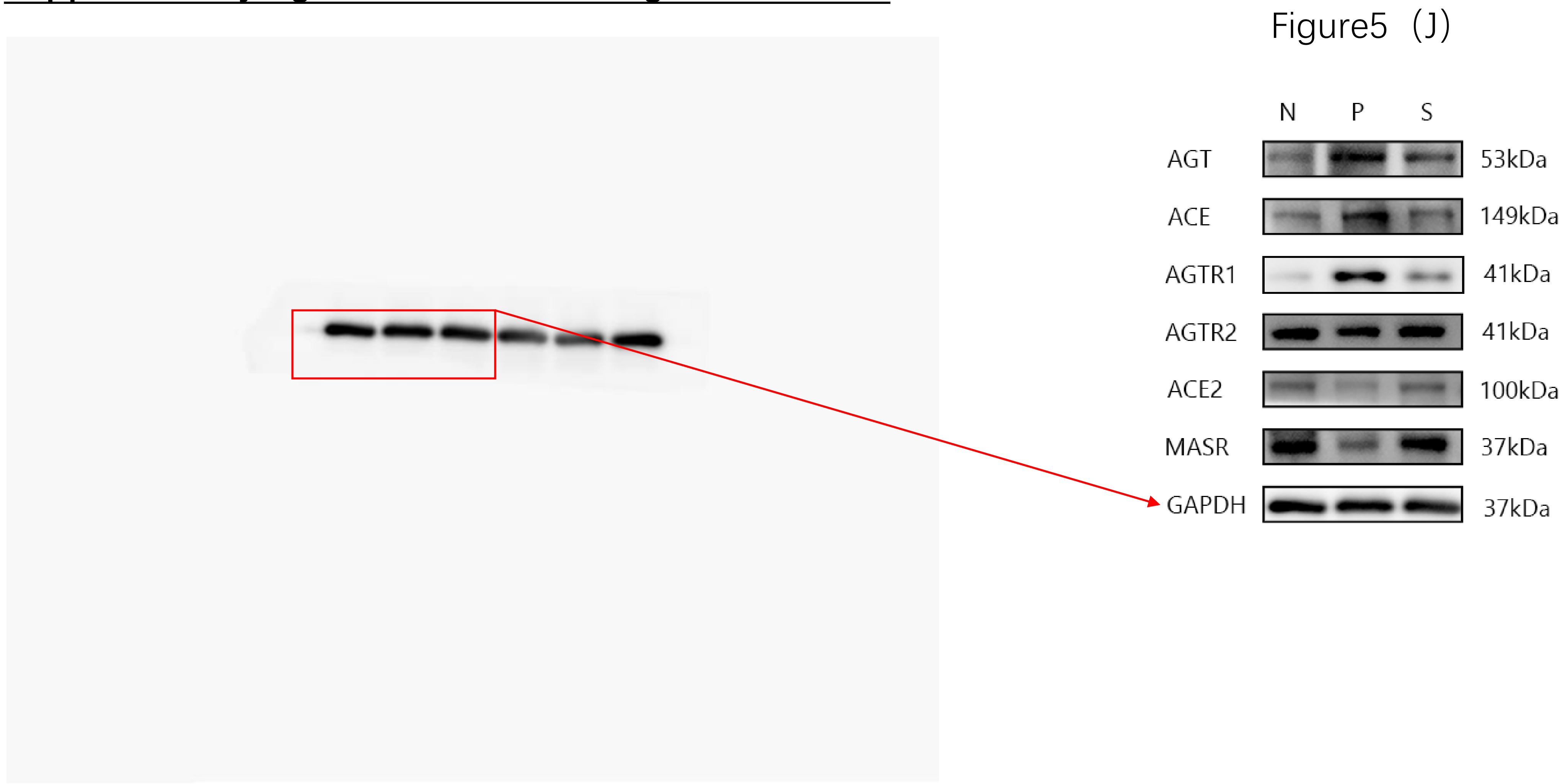

Supplementary figure13.Western Blot gels of BAX

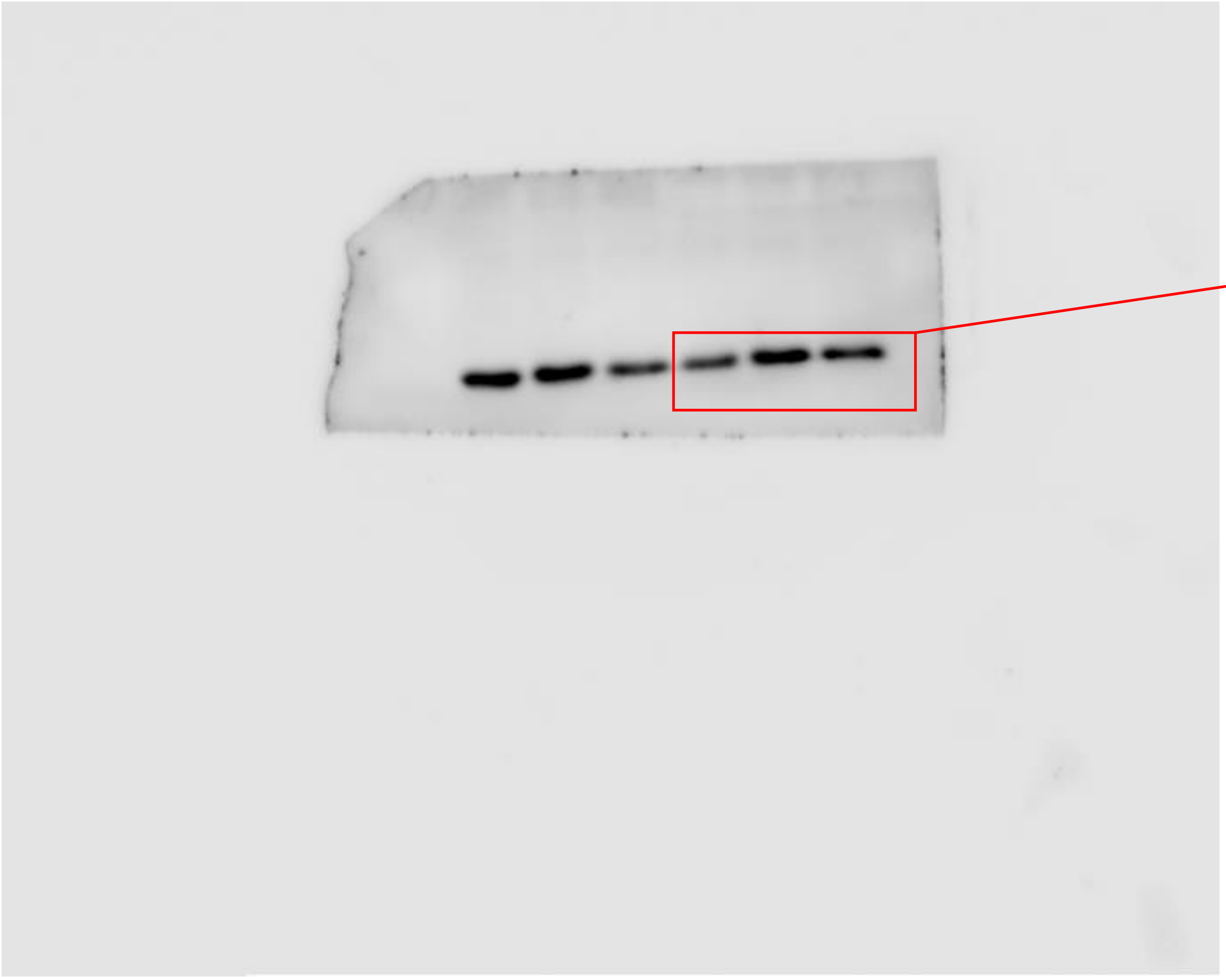

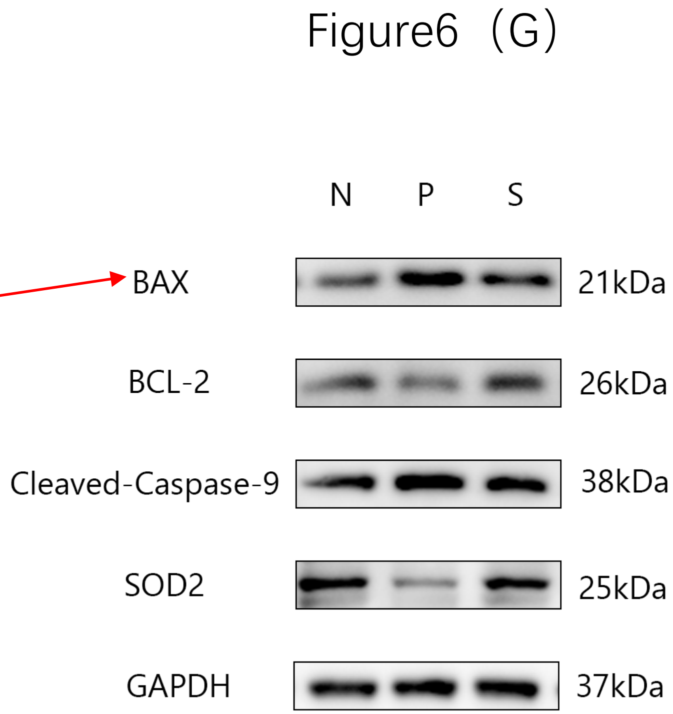

Supplementary figure14.Western Blot gels of BCL-2

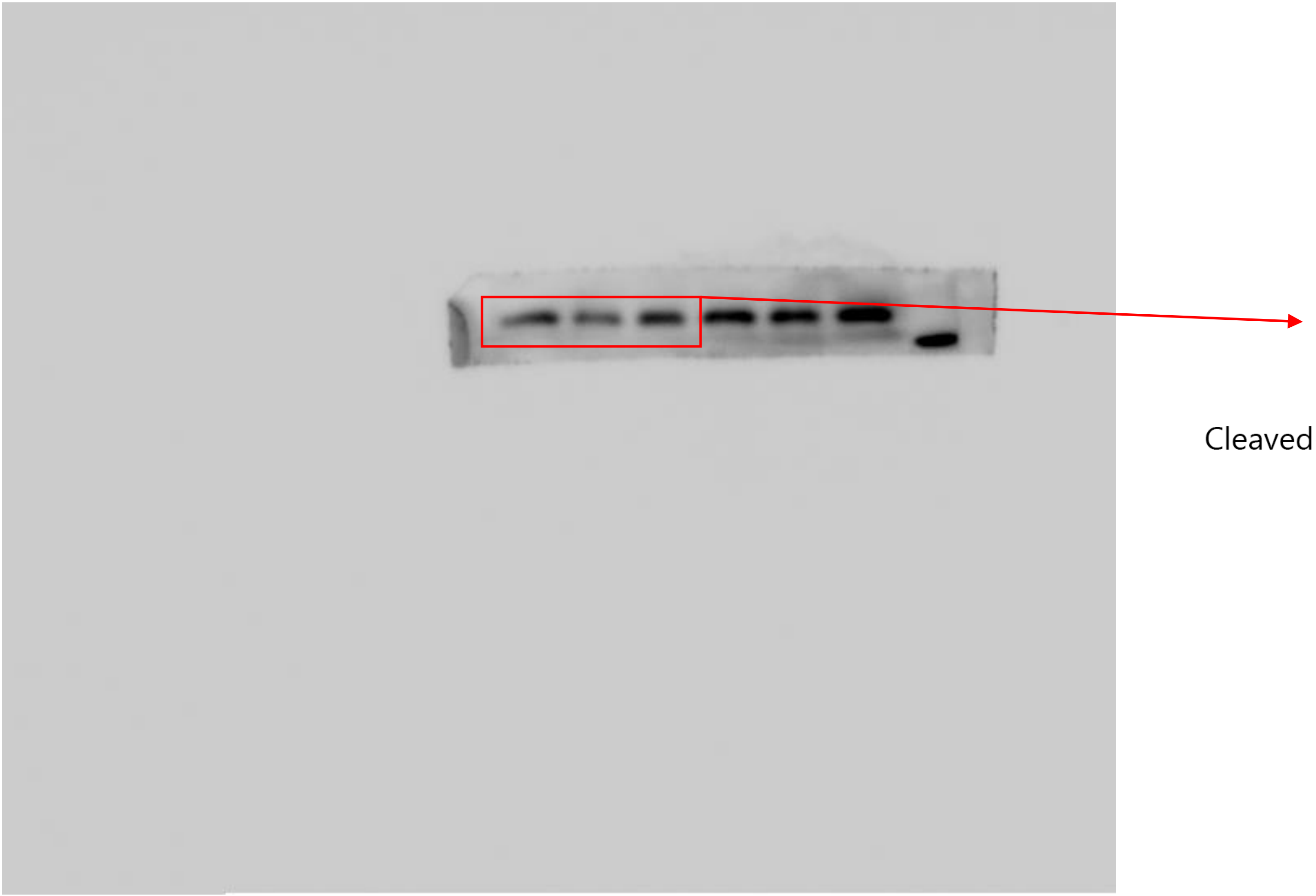

Figure6（G)

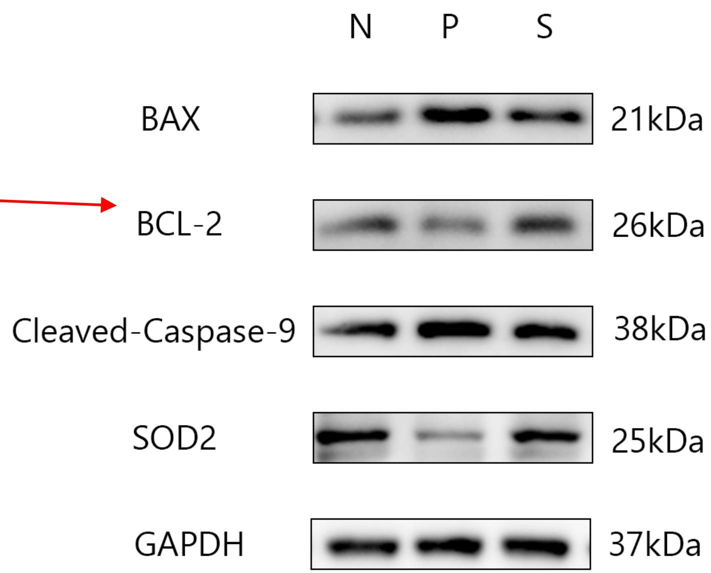

<u>**Supplementary figure15.Western Blot gels of Cleaved-Caspase-9**</u>

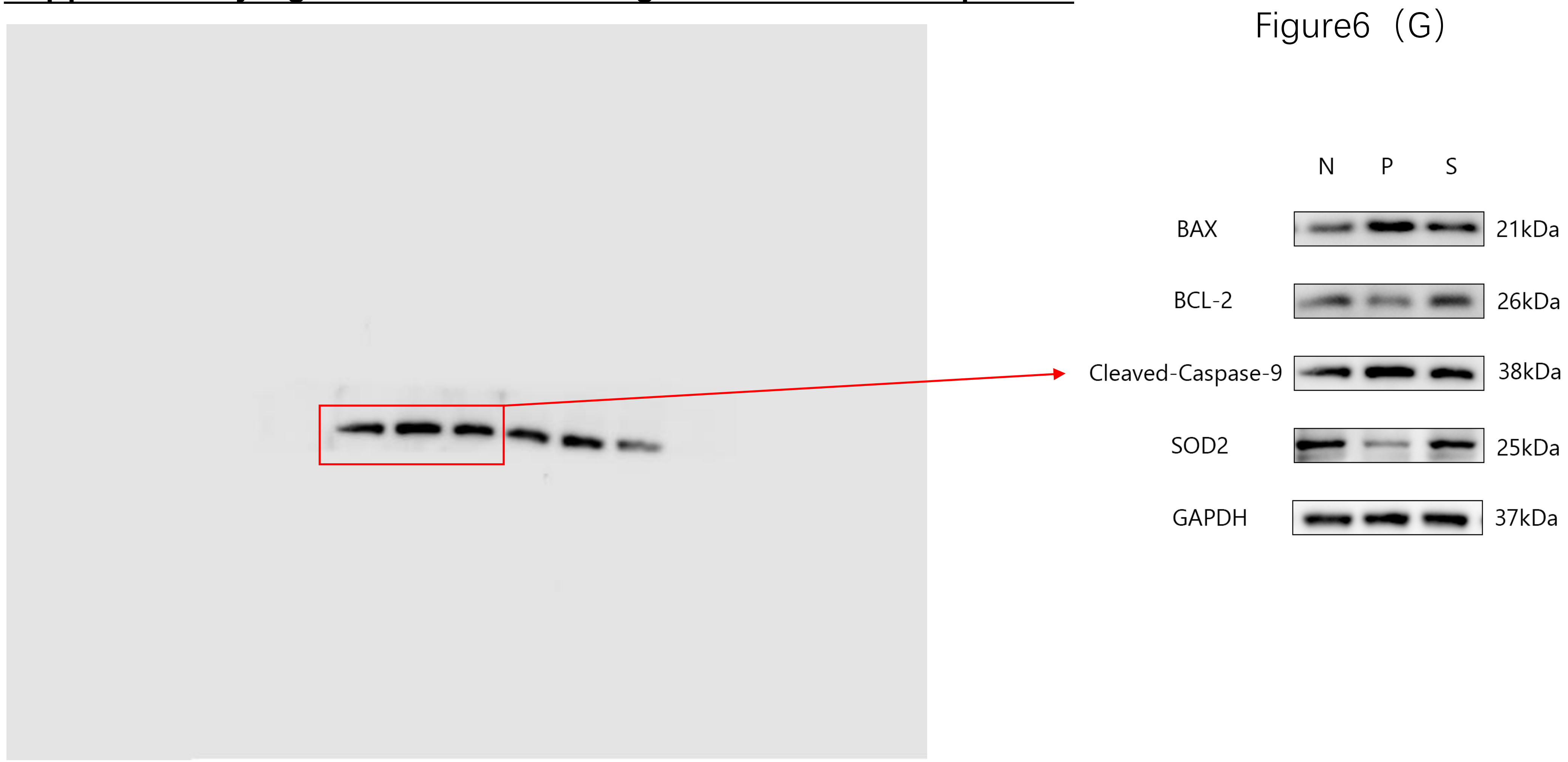

Supplementary figure16.Western Blot gels of SOD2

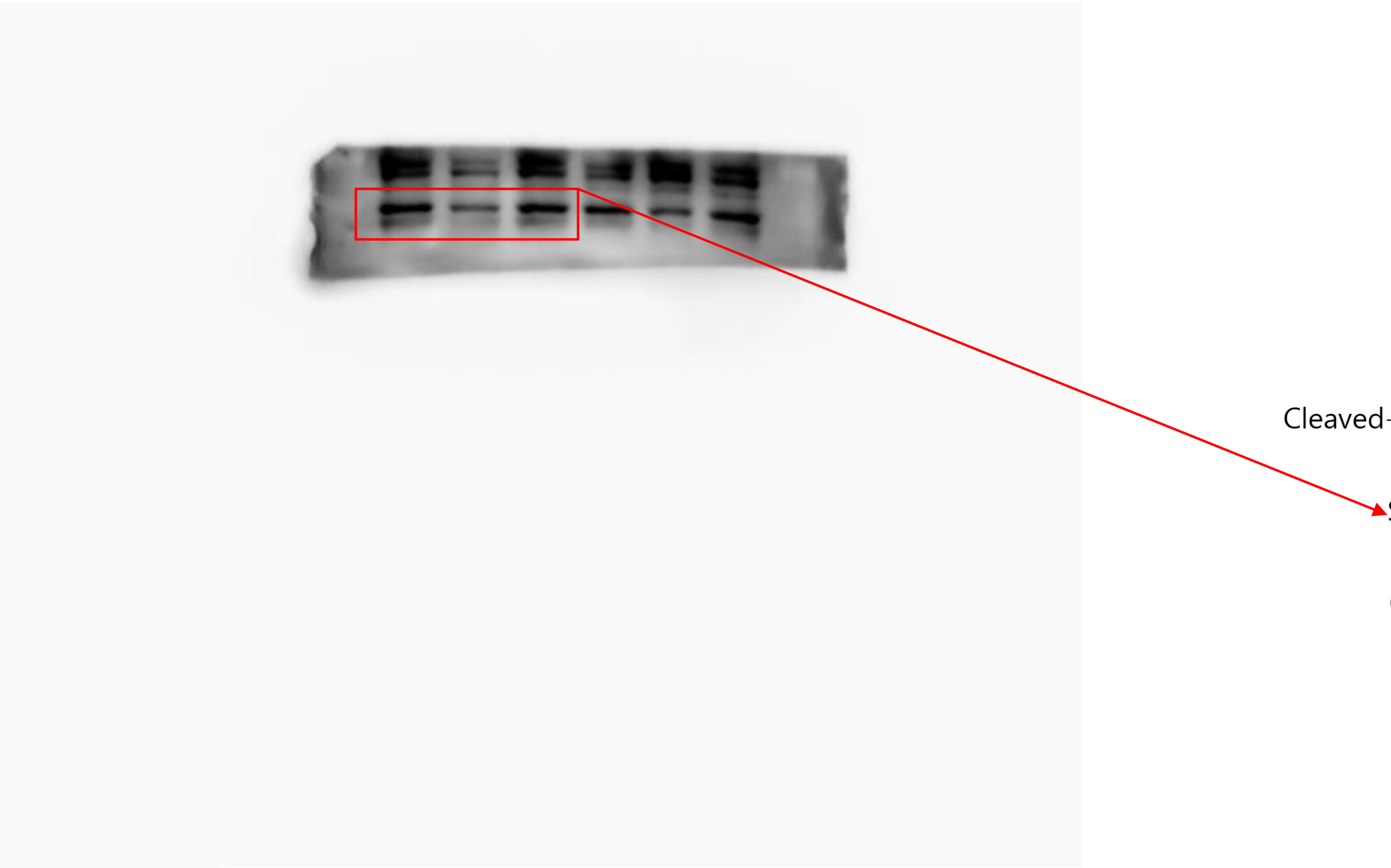

Figure6 （G）

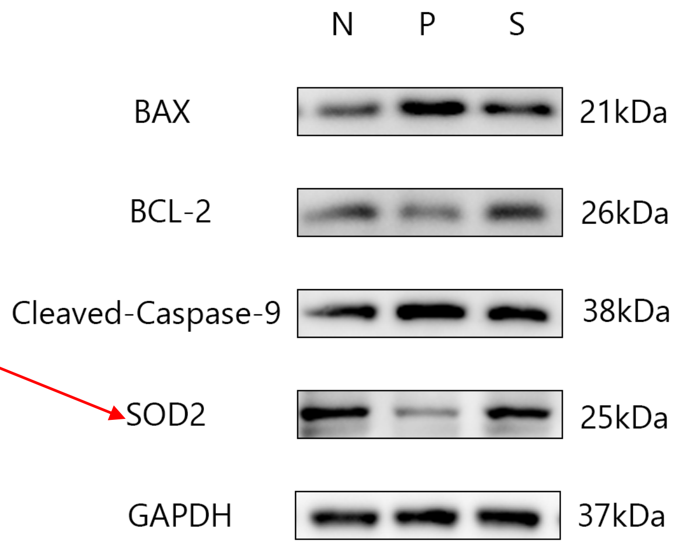

Supplementary figure17.Western Blot gels of GAPDH

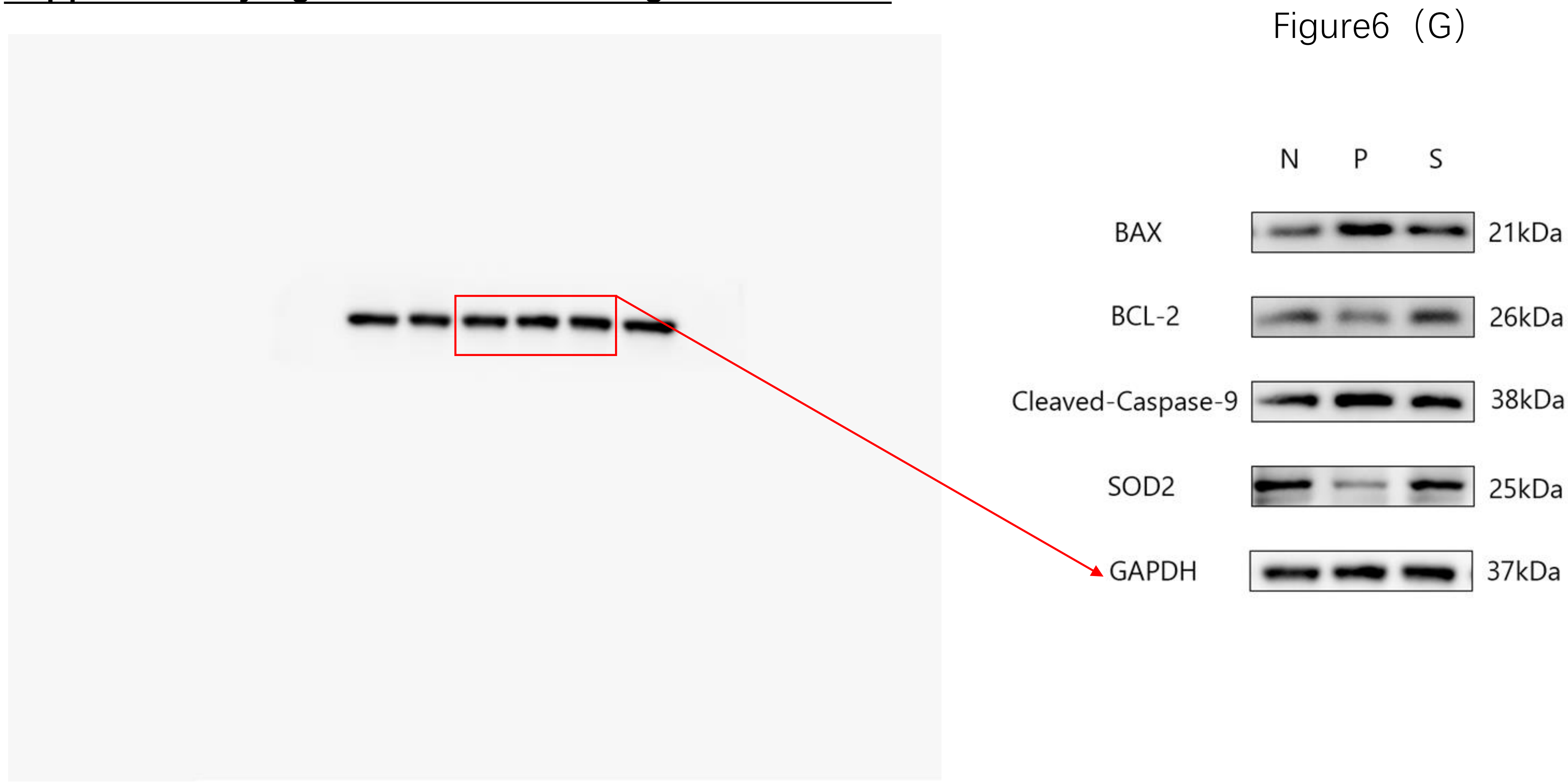

Supplementary figure18.Western Blot gels of AGT

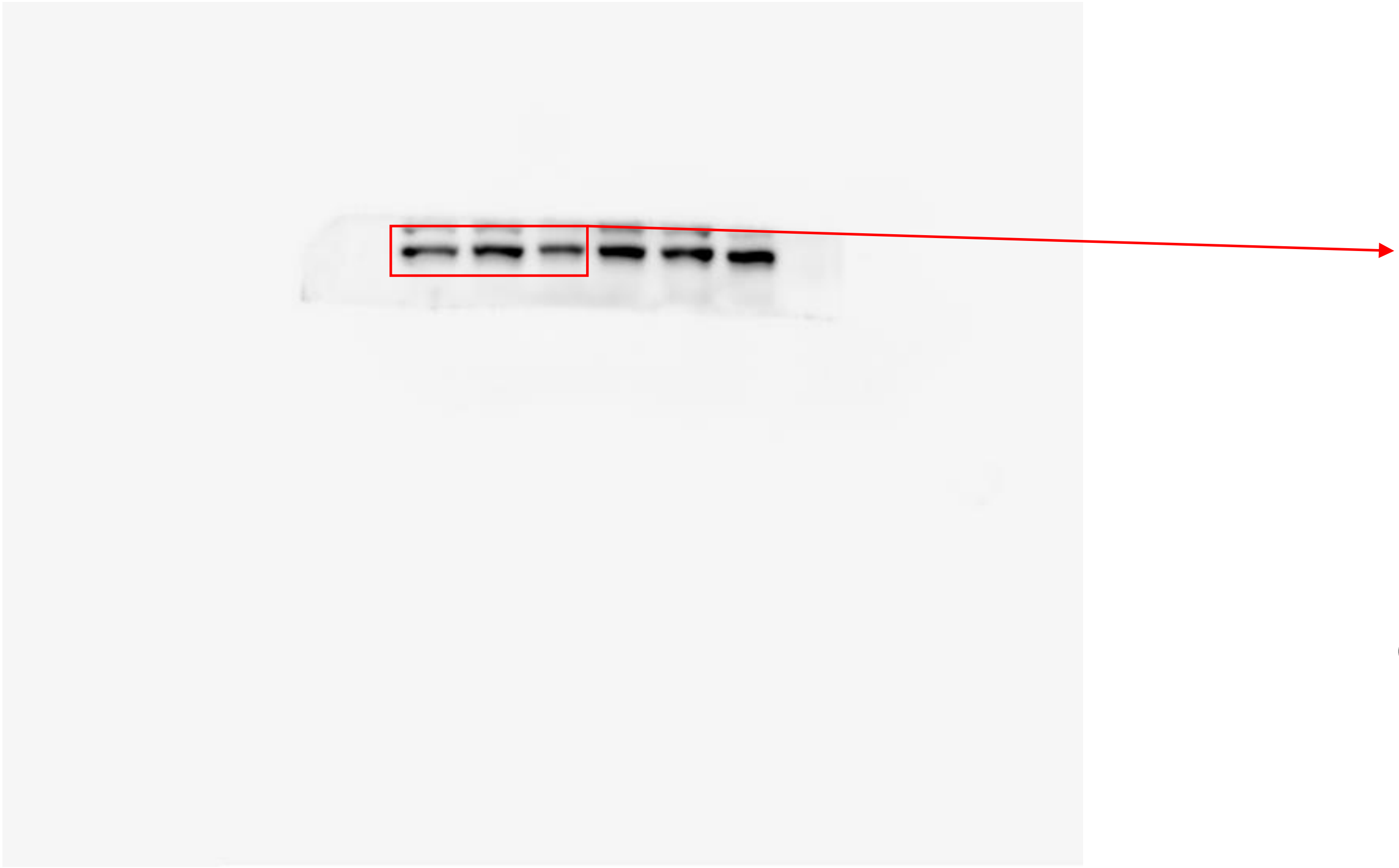

Figure7 （E）

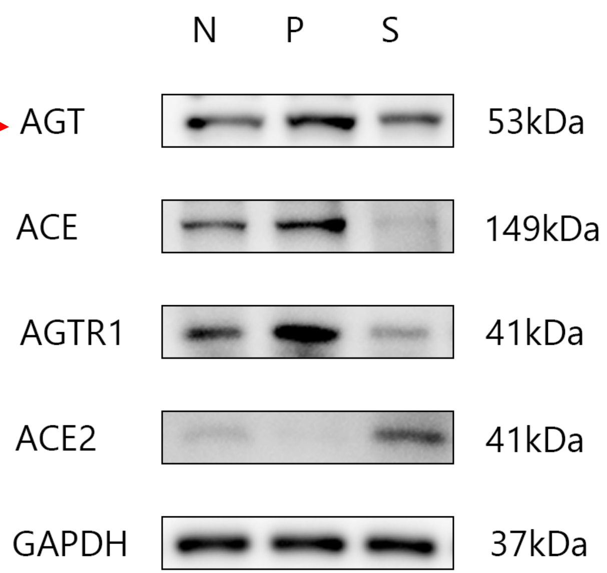

Supplementary figure19.Western Blot gels of ACE

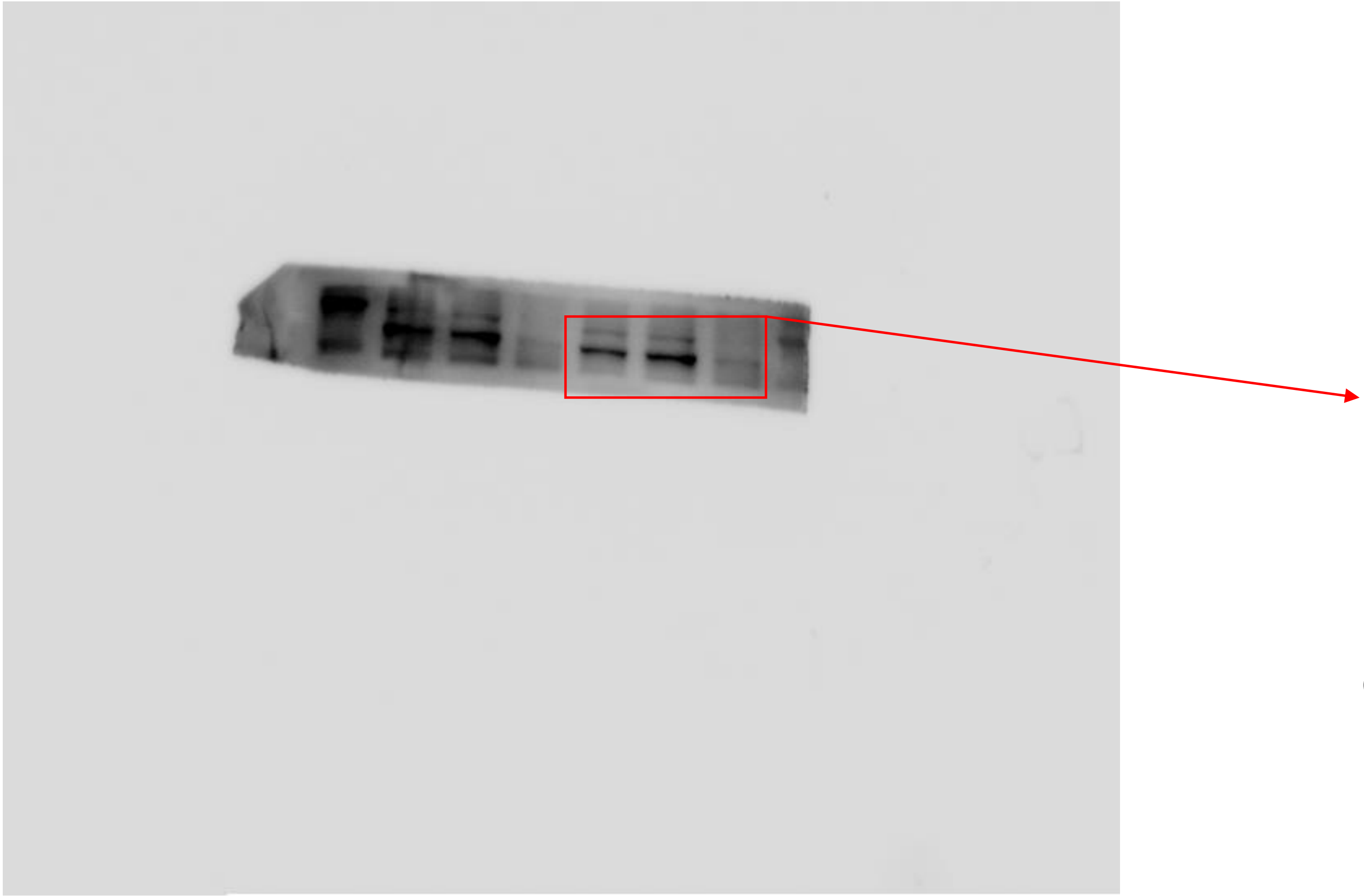

Figure7 (E)

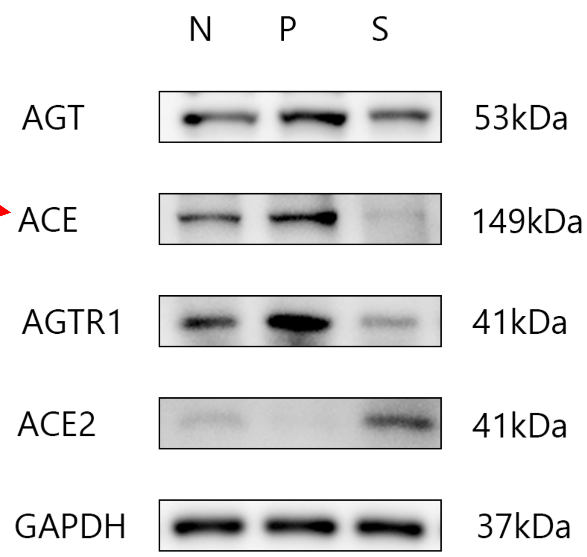

Supplementary figure20.Western Blot gels of AGTR1

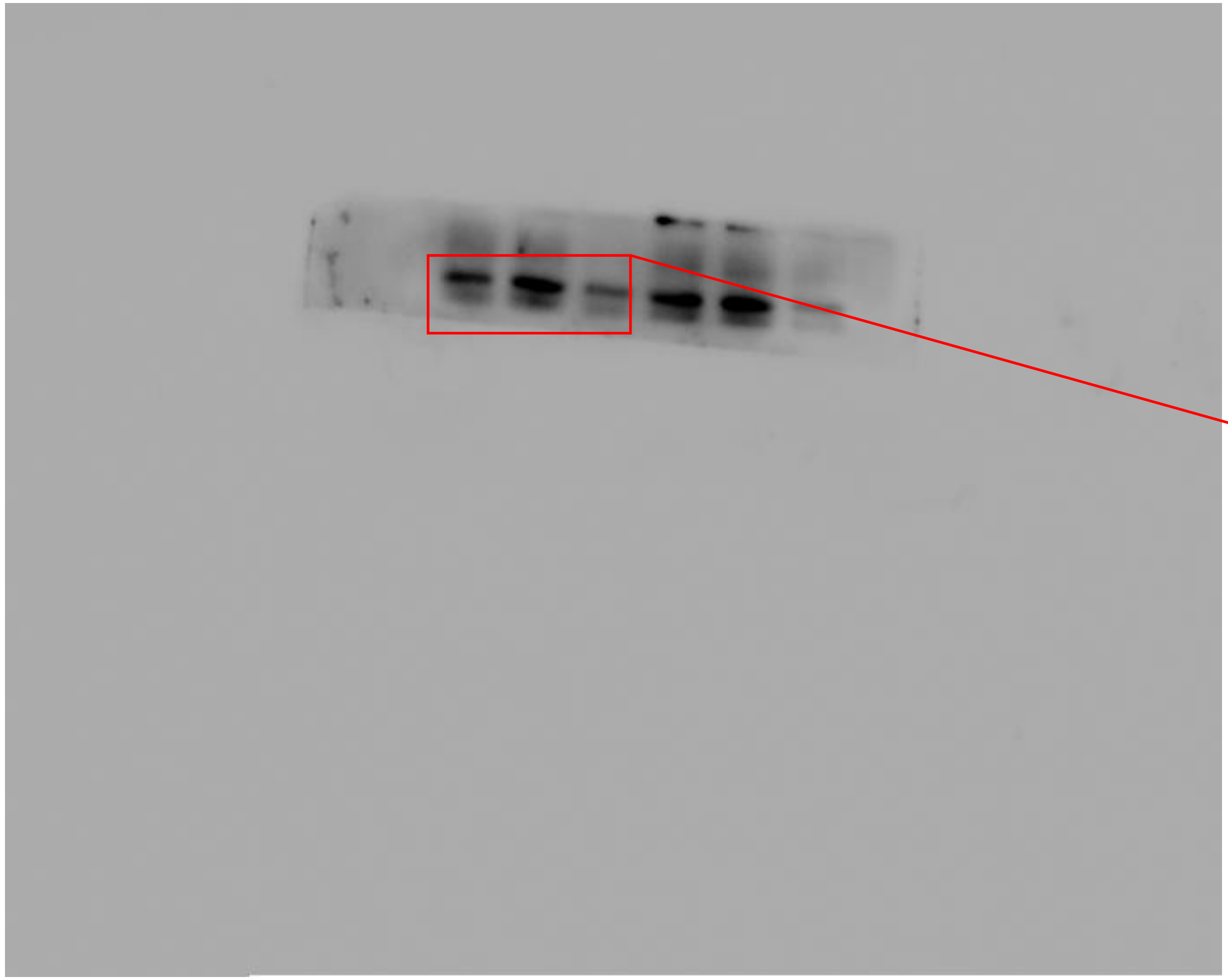

Figure7 (E)

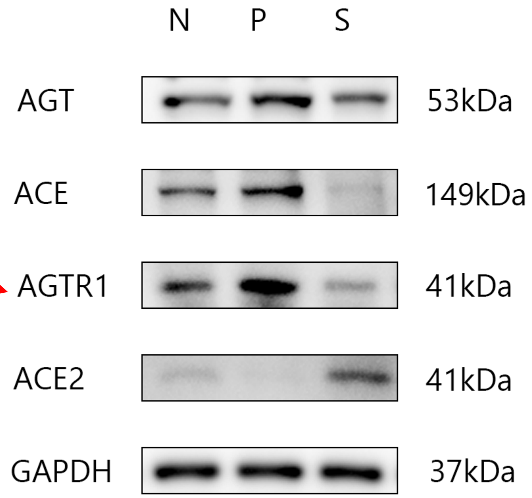

Supplementary figure21.Western Blot gels of ACE2

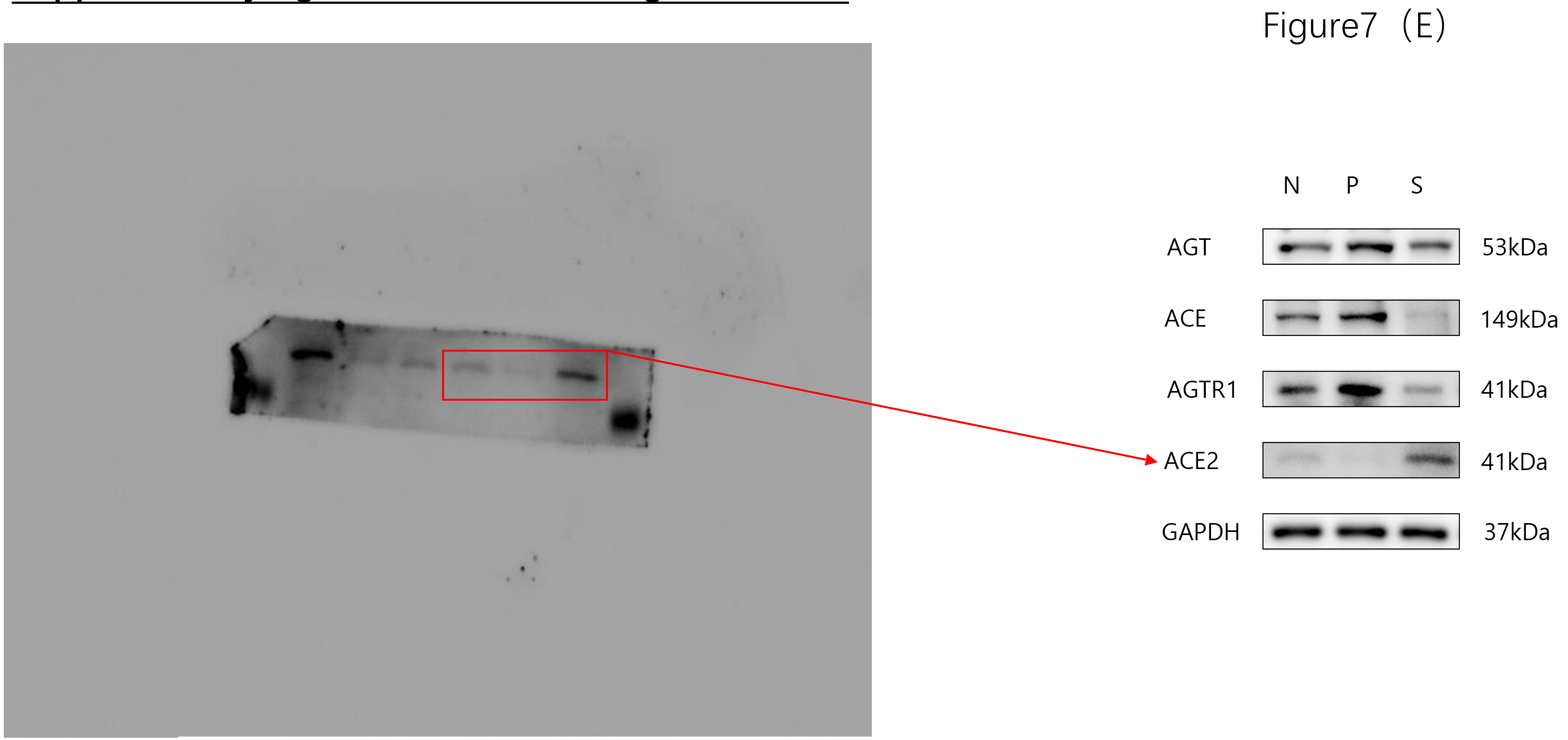

Supplementary figure22.Western Blot gels of GAPDH

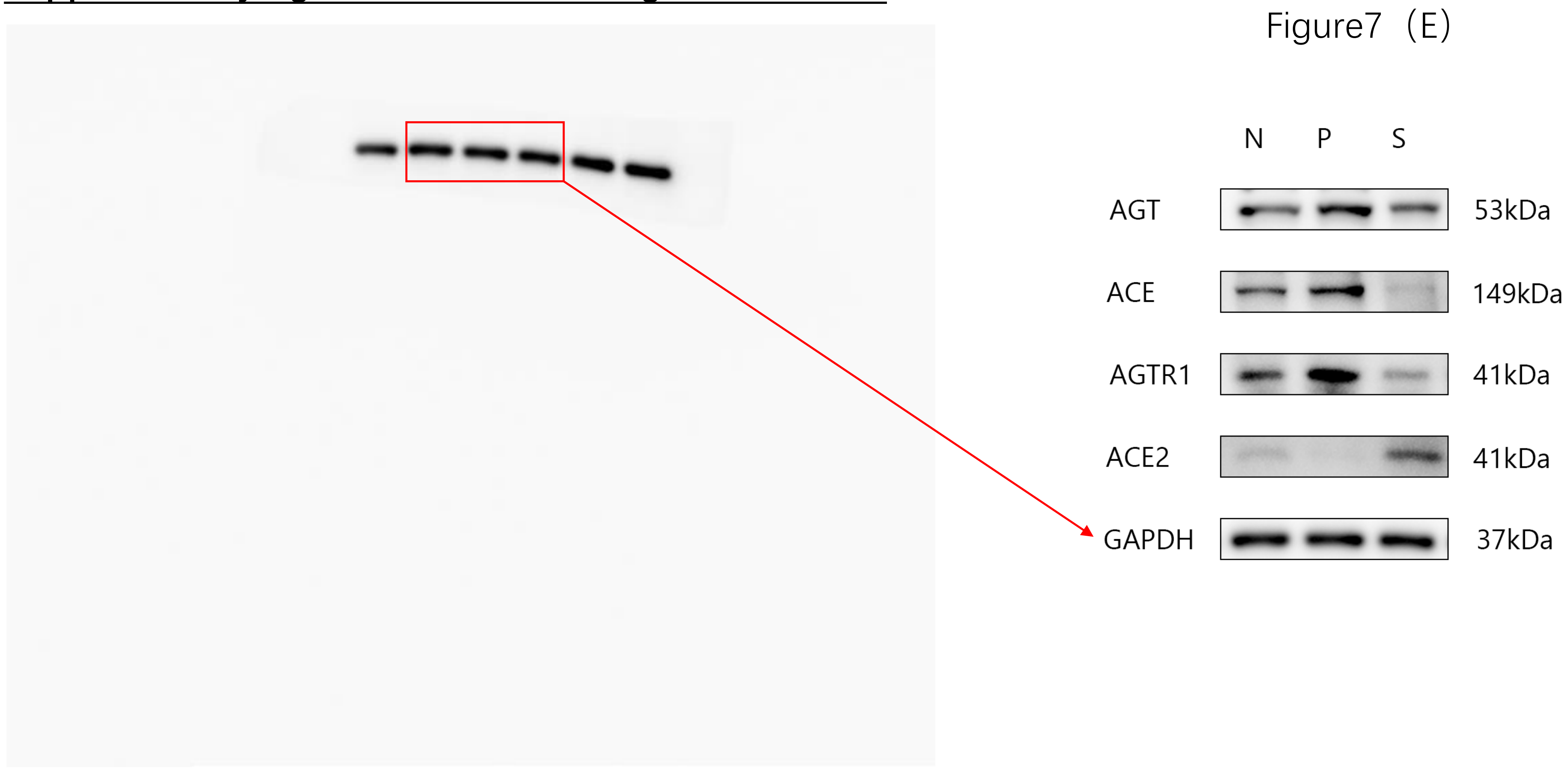

Supplement: Supplementary file 1 — Additional file 1. [file 13048_2023_1337_MOESM1_ESM.pdf]
